# Efficacy and safety of gene therapy with onasemnogene abeparvovec in children with spinal muscular atrophy in the D-A-CH-region: a population-based observational study

**DOI:** 10.1016/j.lanepe.2024.101092

**Published:** 2024-10-07

**Authors:** Claudia Weiß, Lena-Luise Becker, Johannes Friese, Astrid Blaschek, Andreas Hahn, Sabine Illsinger, Oliver Schwartz, Günther Bernert, Maja von der Hagen, Ralf A. Husain, Klaus Goldhahn, Janbernd Kirschner, Astrid Pechmann, Marina Flotats-Bastardas, Gudrun Schreiber, Ulrike Schara, Barbara Plecko, Regina Trollmann, Veronka Horber, Ekkehard Wilichowski, Matthias Baumann, Andrea Klein, Astrid Eisenkölbl, Cornelia Köhler, Georg M. Stettner, Sebahattin Cirak, Oswald Hasselmann, Angela M. Kaindl, Sven F. Garbade, Jessika Johannsen, Andreas Ziegler, SMArtCARE and Swiss-Reg-NMD study group, Petra Baum, Manuela Baumgartner, Astrid Bertsche, Markus Blankenburg, Jonas Denecke, Marcus Deschauer, Matthias Eckenweiler, Tobias Geis, Klaus Goldhahn, Martin Groß, René Günther, Tim Hagenacker, Eckard Hamelmann, Ralf A. Husain, Christoph Kamm, Birgit Kauffmann, Jan Christoph Koch, Wolfgang Löscher, Albert Ludolph, Pascal Martin, Alexander Mensch, Gerd Meyer zu Hörste, Christoph Neuwirth, Susanne Petri, Manuel Pühringer, Imke Rathmann, Dorothee Schäfer, Mareike Schimmel, Bertold Schrank, Olivia Schreiber-Katz, Anette Schwerin-Nagel, Martin Smitka, Meike Steinbach, Elisabeth Steiner, Johannes Stoffels, Manuela Theophil, Raffi Topakian, Matthias Türk, Matthias Vorgerd, Maggie C. Walter, Markus Weiler, Gert Wiegand, Gilbert Wunderlich, Claudia Diana Wurster, Daniel Zeller, Moritz Metelmann, Fiona Zeiner, Veronika Pilshofer, Mika Rappold, Josefine Pauschek, Christof Reihle, Annette Karolin Homma, Paul Lingor, Bettina Henzi, Elisabeth Steiner, Tabea Reinhardt, Dorothea Holzwarth, Wolfgang Wittmann, Manuela Theophil, Stefan Kappel, Maren Freigang, Benjamin Stolte, Kyriakos Martakis, Georg Classen, Doris Roland-Schäfer, Daniela Steuernagel, Hans Hartmann, Sophie Fischer, Marieke Wermuth, Mohamad Tareq Muhandes, Anna Hotter, Zeljko Uzelac, Steffen Naegel, Sarah Wiethoff, Nathalie Braun, Bogdan Bjelica, Heike Kölbel, Daniela Angelova-Toshkina, Bernd Wilken, Alma Osmanovic, Barbara Fiedler, Barbara Plecko, Maike Tomforde, Thomas Voelkl, Arpad von Moers, Petra Müller, Bettina Behring, Anne Güttsches, Peter Reilich, Wolfgang Wick, Corinna Stoltenburg, Simon Witzel, Julia Bellut, Georg Friedrich Hoffmann, Wolfgang Löscher, Kathrin Mörtlbauer, Alexandra Ille, Michael Schroth, Joenna Driemeyer, Luisa Semmler, Cornelia Müller, Katharina Dörnbrack, Manuel Pühringer, Michael Zemlin, Stephanie Geitmann, Hanna Sophie Lapp, Svenja Brakemeier, Tascha Gehrke, Klearchos Ntemiris, Nadja Kaiser, Sabine Borowski, Barbara Ramadan, Ulf Hustedt, Tobias Baum, Simon Witzel, Ilka Schneider, Esra Akova-Oztürk, Katharina Vill, Zylfie Dibrani, Camilla Wohnrade, Adela Della-Marina, Lisa Jung, Timo Deba, Joachim Zobel, Jens Schallner, Christina Kraut, Klaus Goldhahn, Peter Vollmann, Stephanie Schüssler, Melanie Roeder, Miriam Hiebeler, Nicole Berberich, Joanna Schneider, Zeljko Uzelac, Brigitte Brauner, Stefan Kölker, Elke Pernegger, Magdalena Gosk-Tomek, Sarah Braun, Deike Weiss, Gerrit Machetanz, Thorsten Langer, Christina Saier, Sandra Baumann, Sabine Hettrich, Gabriel Dworschak, Katharina Müller-Kaempffer, Isabelle Dittes, Andreas Thimm, Lisa Quinten, Kristina Albers, Andrea Bevot, Christa Bretschneider, Johannes Dorst, Thomas Kendzierski, Iris Hannibal, Jasmin Bischofberger, Tilman Riesmeier, Andrea Gangfuß, Eva Johann to Settel, Michael Grässl, Susan Fiebig, Carmen Hollerauer, Lea Seeber, Ina Krahwinkler, Irene Lange, Federica Montagnese, Marcel Mann-Richter, Alexandra Wagner, Johannes Dorst, Christine Leypold, Afshin Saffari, Elmecker Anna, Anna Wiesenhofer, Eva-Maria Wendel, Paula-Sophie Steffens, Sabine Wider, Adrian Tassoni, Andrea Dall, Franziska Busch, Daniela Zeisler, Maria Wessel, Jaqueline Lipka, Andrea Hackemer, Loreen Plugge, Eva Jansen, Erdmute Roth, Joachim Schuster, Anna Koelsch, Birgit Warken-Madelung, Michaela Schwippert, Britta Holtkamp, Katja Köbbing, Sander Claeys, Sandy Foerster, Daniela Zeisler, Simone Thiele, Heidi Rochau-Trumpp, Annette George, Joachim Schuster, Moritz Niesert, Tanja Neimair, Katia Vettori, Julia Haverkamp, Jila Taherpour, Juliane Hug, Franziska Wenzel, Christina Bant, Ute Baur, Kathrin Bühner, Melina Schlag, Lena Ruß, Hanna Küpper, Anja Müller, Kurt Wollinsky, Therese Well, Antonia Leinert, Barbara Andres, Heymut Omran, Nicole Claus, Kathrin Bühner, Anna Hagenmeyer, Marion Schnurr, Vladimir Dukic, Albert Christian Ludolph, Sabine Specht, Verena Angermair, Anna Hüpper, Daniela Banholzer, Sabine Stein, Tim Kampowski, Marion Richmann, Sylke Nicolai, Omar Atta, Birgit Meßmer, Heike de Vries, Elisabeth Rotenfusser, Alma Oscmanovic, Isabelle Renger, Hélène Guillemot, Ilka Lehnert, Mike Grünwedel, Laura Grimm, Guido Stocker, Angela M. Kaindl, Kurt Wollinsky, Annegret Hoevel, Theresa Stadler, Michal Fischer, Sibylle Vogt, Axel Gebert, Maggie C. Walter, Susanne Goldbach, Tim Hagenacker, Janbernd Kirschner, Hanns Lochmüller, Wolfgang Müller-Felber, Astrid Pechmann, Ulrike Schara-Schmidt, Kristina Probst-Schendzielorz, Annina Lang, Maren Nitzsche, Olivia Schreiber-Katz, Julie Hammer, Katharina Müller-Kaempfer, Corinna Wirner-Piotrowski, Lieske van der Stam, Anke Bongartz, Susanne Goldbach, Hanns Lochmüller, Cornelia Enzmann, Joël Fluss, Elea Galiart, Bettina Henzi, David Jacquier, Dominique Baumann Metzler, Anne Tscherter, Kristina Probst-Schendzielorz

**Affiliations:** a Charité–Universitätsmedizin Berlin, Department of Pediatric Neurology, Augustenburger Platz 1, Berlin 13353, Germany; b Charité–Universitätsmedizin Berlin, Center for Chronically Sick Children, Augustenburger Platz 1, Berlin 13353, Germany; c Charité–Universitätsmedizin Berlin, Institute of Cell Biology and Neurobiology, Augustenburger Platz 1, Berlin 13353, Germany; d Department of Child Neurology, University Hospital, Rudolf-Buchheim-Str. 8, Gießen 35392, Germany; e Clinic for Pediatric Kidney-, Liver- and Metabolic Diseases, Hannover Medical School, Carl-Neuberg Str. 1, Hannover 30625, Germany; f Department of Pediatric Neurology, University Hospital, Albert-Schweitzer-Strasse 33, Münster, Germany; g Department of Pediatrics, Klinik Favoriten, Kundratstr. 3, Vienna 1100, Austria; h Department of Neuropediatrics, Medizinische Fakultät Carl Gustav Carus, Technische Universität Dresden, Fetscherstr. 74, Dresden 01307, Germany; i Department of Neuropediatrics, Jena University Hospital, Bachstr. 18, Jena 07743, Germany; j Department of Pediatrics and Neuropediatrics, DRK Klinikum Westend, Spandauer Damm 130, Berlin 14050, Germany; k Department of Neuropediatrics and Muscle Disorders, Medical Center – University of Freiburg, Faculty of Medicine, University of Freiburg, Heiliggeist-Str. 1, Freiburg 79106, Germany; l University Hospital Homburg, Department of Pediatric Neurology, Kirrberger Str. 100, Homburg 66421, Germany; m Klinikum Kassel, Department of Pediatric Neurology, Mönchebergstr. 41-43, Kassel 34125, Germany; n Department of Pediatric Neurology, Centre for Neuromuscular Disorders, Center for Translational Neuro and Behavioral Sciences, University Duisburg-Essen, Germany; o Department of Pediatrics and Adolescent Medicine, Division of General Pediatrics, Medical University Graz, Auenbruggerplatz 2, Graz 8036, Austria; p Department of Pediatrics, Division of Pediatric Neurology, Friedrich-Alexander University of Erlangen-Nürnberg, Maximiliansplatz 2, Erlangen 91054, Germany; q Department of Pediatric Neurology, University Children’s Hospital, Hoppe-Seyler-Str. 1, Tübingen 72076, Germany; r Department of Paediatrics and Adolescent Medicine, Division of Paediatric Neurology, University Medical Centre Göttingen, Georg August University Göttingen, Germany; s Department of Pediatrics I, Division of Pediatric Neurology, Medical University Innsbruck, Innsbruck, Austria; t Division of Neuropediatrics, Development and Rehabilitation, Department of Pediatrics, Inselspital, Bern University Hospital, University of Bern, Bern, Switzerland; u Department of Paediatrics and Adolescent Medicine, Johannes Kepler University Linz, Kepler University Hospital, Krankenhausstrasse 26-30, Linz 4020, Austria; v Bochum Department of Neuropediatrics, University Children’s Hospital, Ruhr-University Bochum, Bochum, Germany; w Neuromuscular Center Zurich and Department of Pediatric Neurology, University Children’s Hospital Zurich, University of Zurich, Steinwiesstrasse 75, Zurich CH-8032, Switzerland; x Ulm University, Department of Pediatrics, Albert-Einstein-Allee 23, Ulm 89081, Germany; y Department of Neuropediatrics, Children’s Hospital of Eastern Switzerland, St. Gallen, Switzerland; z Heidelberg University, Medical Faculty Heidelberg, Center for Pediatric and Adolescent Medicine, Department I, Division of Pediatric Neurology and Metabolic Medicine, Im Neuenheimer Feld 430, Heidelberg 69120, Germany; aa University Medical Center Hamburg-Eppendorf, Department of Pediatrics, Martinistr. 52, Hamburg 20246, Germany; ab Department of Child Neurology, University Hospital Bonn, Venusberg-Campus 1, Bonn 53127, Germany; ac Department of Pediatric Neurology and Developmental Medicine, Ludwig Maximilian University of Munich (LMU), Hauner Children’s Hospital, Lindwurmstr. 4, Munich 80337, Germany

**Keywords:** Spinal muscular atrophy, Gene addition therapy, SMA, Onasemnogene abeparvovec, Gene therapy, Zolgensma

## Abstract

**Background:**

Real-world data on gene addition therapy (GAT) with onasemnogene abeparvovec (OA), including all age groups and with or without symptoms of the disease before treatment are needed to provide families with evidence-based advice and realistic therapeutic goals. Aim of this study is therefore a population-based analysis of all patients with SMA treated with OA across Germany, Austria and Switzerland (D-A-CH).

**Methods:**

This observational study included individuals with Spinal Muscular Atrophy (SMA) treated with OA in 29 specialized neuromuscular centers in the D-A-CH-region. A standardized data set including WHO gross motor milestones, SMA validated motor assessments, need for nutritional and respiratory support, and adverse events was collected using the SMArtCARE registry and the Swiss-Reg-NMD. Outcome data were analyzed using a prespecified statistical analysis plan including potential predictors such as age at GAT, *SMN2* copy number, past treatment, and symptom status.

**Findings:**

343 individuals with SMA (46% male, 54% female) with a mean age at OA of 14.0 months (range 0–90, IQR 20.0 months) were included in the analysis. 79 (23%) patients were clinically presymptomatic at the time of treatment. 172 (50%) patients received *SMN2* splice-modifying drugs prior to GAT (risdiplam: n = 16, nusinersen: n = 154, both: n = 2). Functional motor improvement correlated with lower age at GAT, with the best motor outcome in those younger than 6 weeks, carrying 3 *SMN2* copies, and being clinically presymptomatic at time of treatment. The likelihood of requiring ventilation or nutritional support showed a significantly increase with older age at the time of GAT and remained stable thereafter. Pre-treatment had no effect on disease trajectories. Liver-related adverse events occurred significantly less frequently up to 8 months of age. All other adverse events showed an even distribution across all age and weight groups.

**Interpretation:**

Overall, motor, respiratory, and nutritional outcome were dependent on timing of GAT and initial symptom status. It was best in presymptomatic children treated within the first six weeks of life, but functional motor scores also increased significantly after treatment in all age groups up to 24 months. Additionally, OA was best tolerated when administered at a young age. Our study therefore highlights the need for SMA newborn screening and immediate treatment to achieve the best possible benefit-risk ratio.

**Funding:**

The SMArtCARE and Swiss-Reg-NMD registries are funded by different sources (see acknowledgements).

**Research in context**

**Evidence before this study**

The pivotal studies START,^1^ STR1VE-US^2^ and STRIVE-EU^3^ that led to an approval of gene therapy with onasemnogene abeparvovec (OA) in patients with spinal muscular atrophy (SMA) included only infants with less than 8.5 kg weight and up to 8 months of age. However, European approval for OA was granted in 2020 for SMA patients with ≤ 3 *SMN2* copies, irrespective of body weight and age,^4^^,^^5^ and the real-world use of OA has been beyond the scientific evidence of clinical trials. To date, postmarketing data are still limited [search date: 1st July 2024, MeSH terms: (“spinal muscular atrophy” OR “sma”) AND (“clinical trial” OR “clinical study” OR “observational study”) AND (“gene therapy” OR “atmp” OR “Onasemnogene Abeparvovec” OR “Zolgensma” OR “AVXS-101”)]. Thus, statistically robust data on effectiveness of OA in large cohorts, especially in neonates and in children older than 8 months, are needed for realistic parental counseling.

**Added value of this study**

This study demonstrates the real-world outcomes of 343 children with SMA treated with OA at a maximum follow-up of 43 months (mean 13.8 ± 9.5), spanning various age and weight ranges with the inclusion of individuals up to 7.5 years and 17.6 kg. Our cohort represents the largest international cohort to date including 179 children older than those treated in clinical trials, as well as 88 patients treated within the first six weeks of life, 79 patients clinically presymptomatic at time of treatment and 172 patients with prior nusinersen and/or risdiplam treatment.

**Implications of all the available evidence**

Outcomes after treatment with OA are better the younger patients are at the time of treatment. Even age-appropriate acquisition of motor milestones can be achieved in children being clinically presymptomatic at the time of treatment. In children who were treated symptomatically within the first 24 months-of-age, functional motor scores still improved significantly, but less profoundly. Benefit-risk-ratio was highest in younger children. This underscores the imperative for SMA newborn screening, facilitating timely treatment intervention, particularly for children at an increased risk of early disease onset.

## Introduction

Spinal muscular atrophy (SMA) is a rare, autosomal-recessive neuromuscular disease characterized by progressive motor neuron loss and subsequent muscle weakness, bulbar and respiratory insufficiency, and often premature death. It is caused by homozygous deletion or mutation of the *SMN1* (*survival motor neuron* 1, MIM 600354) gene resulting in SMN protein deficiency. The paralogous *SMN2* is a disease modifier gene, as higher *SMN2* copy numbers are generally associated with a milder phenotype.^6^ Since 2017, two *SMN2*-splice-modifying therapies (nusinersen and risdiplam) and one gene addition therapy (GAT, onasemnogene abeparvovec (OA)) have been approved.^7^ OA delivers an additional copy of the *SMN1* gene via an adeno-associated virus serotype 9 (AAV9) vector and is administered intravenously as a one-off-treatment. The phase I (START)^1^ and Phase III (STR1VE-US^2^ and STR1VE-EU^3^) trials demonstrated improvement of survival without permanent ventilation and motor development in symptomatic infants with SMA type 1 and 2 *SMN2* copies treated with OA at < 8.5 kg and up to 8 months of age.^1^ Long-term follow-up confirmed sustained efficacy of OA in both symptomatically and presymptomatically treated patients and a favorable safety profile with a median follow-up-time of 5.2 years.^8^ The European label for OA goes beyond the evidence gained from clinical trials by granting approval for treating individuals with SMA with ≤3 SMN2 copies irrespective of body weight and age in 2020.^4^^,^^5^ Hence, the collection of evidence on effectiveness and safety of OA in large cohorts, particularly in patients with clinical characteristics beyond the scope of clinical trials, becomes increasingly crucial. Closing the current knowledge gap on OA is essential for counseling of caregivers with respect to its individual benefit-risk profile given that two alternative therapies are available.

Here, we report effectiveness and safety data from a heterogeneous cohort of 343 patients with SMA who were treated with OA, including specific subgroups of patients such as infants identified by newborn screening and patients treated ≥2 years of age. The size of our cohort allows a comprehensive analysis of factors that may influence outcome such as age at treatment, *SMN2* copy number, symptom status and baseline motor function at the time of therapy.

## Methods

### Study design and participants

The disease-specific patient registries SMArtCARE^9^ and Swiss-Reg-NMD provide a systematic, prospective collection of data on motor function and milestones, respiratory and nutritional support, and treatment-related side effects in SMA patients on and off treatment. In this protocol-based registry study, we collected data from individuals with 5q-SMA who were treated with OA in 29 specialized neuromuscular centers in Germany, Austria, and Switzerland between September 19th, 2019, and January 27th, 2023, including all ages, individuals with homozygous and compound heterozygeous *SMN1*-variants, two and three *SMN*2 copy numbers, and children with previous treatment with nusinersen and/or risdiplam as well as untreated children. Patients’ legal guardians had to consent to pseudonymized data collection within either the SMArtCARE registry (Ethics Committee Freiburg-Germany, no. EA56/18) or the Swiss-Reg-NMD (Cantonal Ethics Committee, Bern, no. 2018-00289). There were no specific inclusion or exclusion criteria for data collection, however patients with either one or four copies of the *SMN2*-gene were excluded from the analysis due to small sample size and off-label use respectively. There was no general minimal follow-up, as we collected safety and baseline data from all patients regardless of follow-up, Mean follow-up time was 13.8 months (SD: 9.5, range 0–43 months). For inclusion in the analysis of CHOP INTEND and HINE, at least one score at baseline and after a minimal follow-up of 6 months was required. As HFMSE and RULM cannot be obtained in infants, no baseline score was required for inclusion in the analysis. Instead, at least two data sets at least 6 months apart were required. Data cut for this study was February 10th, 2023.

Safety data was collected in terms of “Adverse Events” (AE) in the SMArtCARE-registry, however there was no specific definition for a collection of these events in a post-marketing-setting. In a second step, physicians were asked whether an AE was related to OA treatment and whether inpatient treatment was necessary. In case of hospital admission an AE was automatically classified as “Serious Adverse Event” (SAE). AE were classified into the following categories: “hepatopathy” (hepatopathy, elevation of liver enzymes), “cardiac events”, “respiratory tract infection” and “other”. Only AE termed as “related to OA” were included in the statistical analysis.

### Procedures before and after GAT

Baseline and follow-up examinations were collected as part of standard of care according to a published German consensus protocol^10^ and the current version of the Summary of Product Characteristics (SmPC,^5^) for OA. OA and prednisolone treatment were managed accordingly. Some children were treated prior to the European Medicines Agency (EMA^5^) and Swissmedic approvals, consistent with the US Food and Drug Administration (FDA) regulatory label.^4^ Two patients with 4 *SMN2* copies received off-label-treatments with OA.

The treating neuromuscular pediatric neurologist determined whether the condition was symptomatic or clinically presymptomatic. Clinical data, genetic results (*SMN1* MLPA, *SMN2* copy number), information on adverse events and on effectiveness were extracted from the two registries. According to the German consensus protocol, survival, respiratory and feeding support, and motor skills including the Children’s Hospital of Philadelphia Infant Test of Neuromuscular Disorders (CHOP INTEND), the Hammersmith Functional Motor Scale-Expanded (HFMSE), the Revised Upper Limb Module (RULM), Hammersmith Infant Neurological Examination (HINE), and the motor milestones independent sitting, standing and walking were assessed before GAT as baseline parameters, on days 30 and 60 and every 4 months thereafter. Detailed information about the outcome measurements is summarized in the Supplementary material.

### Statistical analysis

A pre-specified statistical analysis plan based on previous publications was used.^10^^,^^11^ For CHOP INTEND, HFMSE, RULM, HINE scores, all patients with at least one baseline score and a second score after GAT were included. Since RULM values are generally not obtained in younger children at baseline, RULM scores were only analyzed descriptively, but no statistical analysis was performed. Statistical analysis was performed using R language version 4.3.1. CHOP INTEND, HFMSE and HINE scores were analyzed with generalized additive (GAM) and linear mixed model (LMM) models. In GAM, the interaction between predictor variable age at GAT (months) and observation period relative to GAT (negative months corresponds to before GAT, positive after GAT) and their combined effect on any of the scores were analyzed. R package ‘mgcv’ version 1.9–0 and ‘itsadug’ version 2.4–1 were used for GAM analysis. For LMM with random intercept, observation period relative to GAT in months were categorized in seven intervals: −6, 0 (GAT), 6, 12, 18, 24, and >30, and the measured score with the smallest time difference to the interval was selected for analysis. Due to sparseness, not all observation periods could be included in LMM analysis. Beside categorized observation period, number of *SMN2* copies (2/3), pre-symptomatic at GAT (no/yes), age at GAT (0–1, 1–8, 8–24, 24–36, and >36 months), and previous medication with nusinersen and/or risdiplam (no/yes) were added as predictor variables, and subject identifiers as random effect. First, a saturated LMM model with all two-way interactions between observation period and all four predictor variables was modeled; a significant two-way interaction (e.g., observation period and past medication) indicated that the time course of a score was moderated by a predictor variable. Then, backward variable selection based on p-values was applied to reduce complexity of the saturated LMM model by removing non-important interaction terms. LMMs were modeled with R package ‘lme4’, version 1.1–34, and function ‘step’ in R package ‘lmerTest’ version 3.1–3 was used for backward variable selection. Post-hoc comparisons were carried out using estimated marginal means with proportional weighting (R package ‘emmeans’, version 1.8–8). For respiratory and nutritional support before and after GAT, McNemar test were applied. Kaplan–Meier estimates and Cox-proportional hazard regression were applied to right censored outcomes (reaching a motor milestone such as independent sitting, standing, and walking, feeding and respiratory support), with predictor variables age at GAT, number of *SMN2* copies and presymptomatic status at GAT. Cox model assumptions were tested by R function ‘cox.zph’ from R package ‘survival’, and all computed Cox models hold the proportional hazards assumption. R package ‘simPH’ version 1.3–13 was used for simulating and plotting relative hazards from Cox regression models. Baseline characteristics, follow-up duration, and influence of age at treatment on functional assessments were analyzed descriptively.

Association between number of adverse events and age at GAT was analyzed with a Poisson regression model. No missing data was imputed, and all sub-analyses were computed with a complete data set with respect to outcome and selected covariates.

### Role of the funding source

None of the funders was involved in the study design, data collection, analysis, interpretation of data or writing the article. None of the funders had any influence on data collection, statistical analyses or interpretation of the results.

## Results

### Cohort

Our cohort consisted of 347 individuals with SMA. Patients with 1 (n = 2) and 4 (n = 2) *SMN2* copies were excluded from statistical analyses due to low number of patients, leaving 343 children for statistical analyses (for baseline characteristics refer to Table 1). 117 infants were identified by newborn screening, furthermore 15 via positive family history and in 2 children diagnosed soon after birth no further information was available. Of these, 30 developed symptoms during the first month-of-age. At the time of treatment, 79/343 children (23%) were classified as presymptomatic (asymptomatic). Before GAT, 154 (44.0%) had received nusinersen, 16 (4.6%) risdiplam, and two patients (0.6%) both. These previous treatments were discontinued prior to GAT. After GAT, two patients received an add-on therapy with risdiplam upon physicians’ decision. Follow-up time was up to 43 months (mean 13.8, IQR 17.0 months), in 169 patients (49.2%) it was ≥24 months.

**Table 1:** Participant baseline characteristics.

Characteristics (n = 343)	
**Sex**	
Female	185 (54)
Male	158 (46)
**SMA type**	
Presymptomatic	79 (23)
1	152 (44)
2	53 (16)
3	5 (1)
***SMN2* copy number**	
2	207 (60)
3	136 (40)
**Pre-treatment**	
Naïve	171 (50)
Nusinersen	154 (45)
Duration, months, mean ± SD (range)	12.0 ± 12.7 (0–55)
Interval to GAT, days, mean ± SD (range)	74.1 ± 45.5 (1–317)
Risdiplam	16 (5)
Duration, months, mean ± SD (range)	3.3 ± 3.0 (0.5–9)
Interval to GAT, days, mean ± SD (range)	20.8 ± 42.8 (1–185)
Nusinersen + risdiplam	2 (0.6)
**GAT treatment details**	
Age at infusion, months, mean ± SD (range)	14.0 ± 15.4 (0–90)
Weight at infusion, kg, mean ± SD (range)	7.7 ± 3.4 (1.7–17.6)
Follow up, months, mean ± SD (range)	13.8 ± 9.5 (1–43)
**Adverse events (AE), n (%)**	123 (36)
Hospitalization	40 (12)
Hepatic AE	93 (27)
Thrombocytopenia	13 (4)
Thrombotic microangiopathy	1 (0.3)
Elevated cardiac enzymes	17 (5)
**Death, n (%)**	6 (1.7)
Age at death, months, mean ± SD (range)	11 ± 10.8 (3–27)
Reasons: Infection/respiratory failure	
Aspiration leading to hypoxia	2
Cardiac arrest	1
Respiratory insufficiency, additional co-morbidities	1
Unknown circumstances	1

Data are number (n) of patients, percentage (%) is given in brackets unless otherwise specified.

### Motor outcome

Motor scores were analyzed with a complete set of covariates, therefore not all observations could be analyzed due to missing values of covariates. CHOP INTEND scores were available in 241 children, HFMSE scores in 133 children, HINE scores in 279 children and RULM scores in 103 children. Descriptive data of longitudinal motor scores categorized in age categories, presymptomatic diagnoses at treatment, *SMN2* copy number, pre-treatment regimes, and sex are summarized in Supplementary Tables S2–S5.

**Fig. 1:**
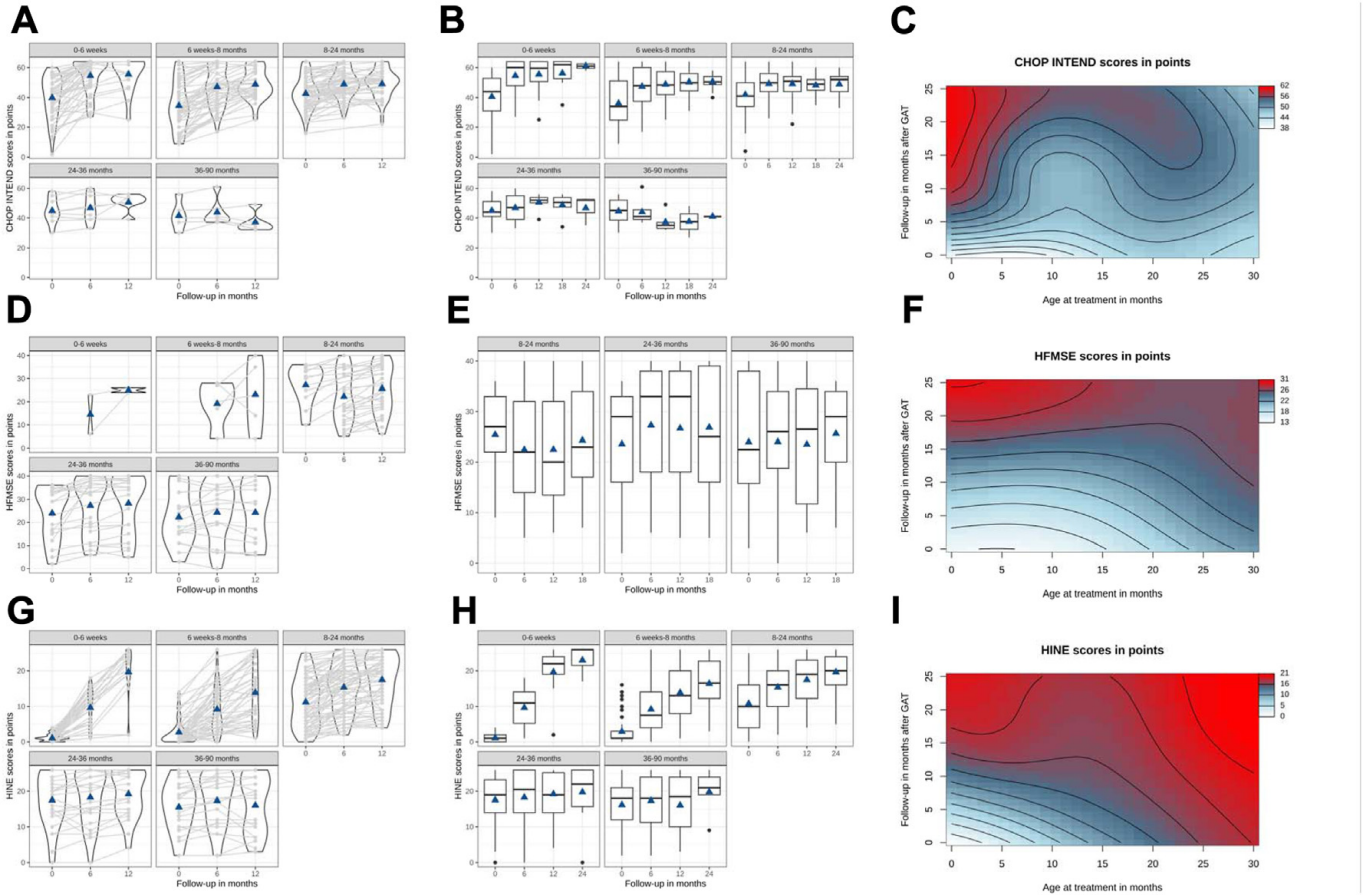
**CHOP INTEND, HFMSE and HINE scores before and after GAT.** Individual course of **(A)** CHOP INTEND, **(D)** HFMSE and **(G)** HINE over 12 months categorized by age groups (linear mixed model, LMM). CHOP INTEND scores increased significantly in all age categories up to 24 months (p < 0.001) and correlated inversely with age at GAT (p < 0.001). Increases in HFMSE scores correlated inversely with age at GAT. Increase in HINE correlated inversely with age at GAT (p < 0.001). Light gray lines depict individual courses, width of figures the number of patients represented and triangles mean values. **(B)** CHOP INTEND, **(E)** HFMSE and **(H)** HINE courses over the observation period in months categorized by age groups at time of GAT (linear mixed model, LMM). CHOP INTEND scores increased significantly in patients treated up to 24 months (all: p < 0.0001), however in patients treated 24–36 months (p = 1.00) and >36 months (p = 1.00), scores did not increase significantly. Score increase correlated inversely with age at GAT, with the highest increase in children treated ≤6 weeks (p < 0.001). Follow-up HFSME scores increased significantly from 6 to 12 months after GAT in patients treated ≤6 weeks and 6 weeks-8 months. Baseline to follow-up HFMSE-scores significantly increased from 8 to 24 and 24–36 months (p < 0.001). In patients treated >36 months, scores did not increase significantly (p = 0.6968). HFSME score increase correlated inversely with the age at GAT. HINE increase significantly in patients treated ≤6 weeks (p < 0.001), 1–8 months (p < 0.001), 8–24 months (p < 0.001) and 24–36 months (p = 0.03684). In patients treated >36 months, scores did not increase significantly (p = 0.1655). Overall age groups, HINE score increases correlated inversely with the age at GAT, with the highest increase in children treated ≤6 weeks (LMM: p < 0.001). Predicted **(C)** CHOP INTEND, **(F)** HFMSE and **(I)** HINE in a Generalized Additive Model (GAM). Wavy lines indicate a change in CHOP INTEND scores, with blue values being lower than red ones. CHOP INTEND, HFMSE and HINE scores were significantly higher over the follow-up period the younger the patients were at GAT (GAM, p < 0.0001 each), and patients treated younger crossed more motor score lines.

CHOP INTEND score increase correlated inversely with age at GAT, with the highest increase in children treated ≤6 weeks (LMM: p < 0.001, Fig. 1A and B, Supplementary Fig. S1A). Scores were significantly higher over the follow-up period the younger the patients were at GAT (GAM, p < 0.001, Fig. 1C). In patients >24 months [24–36 months (LMM, p = 1.00) and >36 months (LMM, p = 1.00)], CHOP INTEND scores did not increase within 12 months after GAT but were stable. Higher CHOP INTEND scores at baseline resulted in higher scores 6 and 12 months after GAT: While most children treated <8 months with a CHOP INTEND score >40 reached scores >60 after 6 months, few of those with a baseline score <40 reached highest scores. In children >8 months at GAT with a baseline CHOP INTEND score >50, most reached scores >60 by 6 months, whereas almost none of those with scores <50 reached highest scores (Supplementary Fig. S4). The same significant influence of the age at GAT could be observed in longitudinal observations of the HINE score (LMM, GAM: p < 0.001, Fig. 1G and H, Supplementary Fig. S1C).

In children <8 months, no baseline investigations were obtained for HFMSE due to test design, therefore only longitudinal effects were included in the statistical analysis. HFSME scores at baseline and longitudinal were mostly obtained in children beyond the age of 8 month. Therefore, statistical comparisons of HFMSE scores were computed from 6 to 12 months after GAT: the increase was statistically significant in patients from 0 to 6 weeks (LMM: p = 0.0024), 1–8 months p = 0.0024) and 8–24 months (LMM: p = 0.0024), but not for 24–36 months (LMM: p = 0.681), and from 36 to 90 months [(LMM: p = 0.793), see Fig. 1D, E, F, Supplementary Fig. S1B]. RULM scores also increased 6 and 12 months after GAT; however, due to small sample size and test, no statistical analysis was performed (Supplementary Fig. S2A–C).

**Fig. 2:**
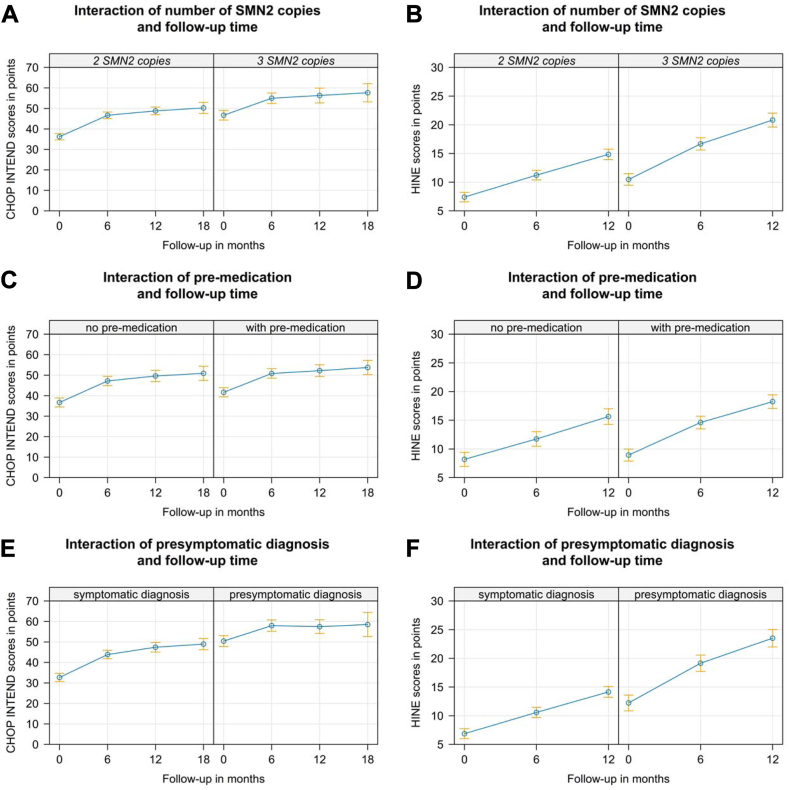
**Effect graphs for CHOP INTEND and HINE in a linear mixed model (LMM).** Effect graph of the interaction between follow-up-time and *SMN2* copy number show generally increased **(A)** CHOP INTEND- and **(B)** HINE-baseline scores in the group with 3 vs. 2 *SMN2-*copies (p < 0.001). However, motor outcome trajectories did not change after GAT. **(C)** Effect graph of the interaction between follow-up-time and pre-medication with either nusinersen or risdiplam show generally increased CHOP INTEND scores in the group with pre-medication (p < 0.001). However, motor outcome trajectories did not change after GAT in comparison to those under pretreatment with either nusinersen or risdiplam (p = 0.254). **(D)** The same effect is seen in HINE scores, however baseline HINE scores are not significantly different in pretreated children vs. therapy naïve ones. **(E)** Effect graph of the interaction between follow-up-time and symptomatic vs. presymptomatic at GAT show generally increased CHOP INTEND scores and baseline (p < 0.001) but not trajectories ((p = 0.09) in the group with a presymptomatic at GAT. CHOP INTEND in presymptomatic children rapidly reaches saturation niveau of >60 points. Therefore, significantly higher baseline HINE-scores scores and HINE outcome trajectories in presymptomatic children are reflected in **(F)**.

In addition to age at GAT, we found a significant effect of *SMN2* copy number for all motor scores (LMM, p < 0.001, for CHOP INTEND and HINE see Fig. 2A and B, Supplementary Tables S6–S8). Children pre-treated with either nusinersen or risdiplam at time of GAT had significantly higher CHOP INTEND- (p < 0.001), but not HINE-scores (p = 0.929) and HFMSE- (p = 0.081); for CHOP INTEND see Fig. 2C–D, Supplementary Tables S6–S8). However, motor outcome trajectories did not change after GAT in comparison to those under pretreatment with either nusinersen or risdiplam (CHOP-INTEND p = 0.254, HFMSE p = 0.223, HINE p = 0.157; Fig. 2C–D, Supplementary Tables S6–S8).

Children treated presymptomatically with GAT (n = 79) had significantly higher scores in all motor function tests regardless of age at treatment for CHOP INTEND (LMM, p < 0.001), HFSME (p = 0.001) and HINE p < 0.001) see Fig. 2E–F, Supplementary Tables S6–S8).

### Milestones

**Fig. 3:**
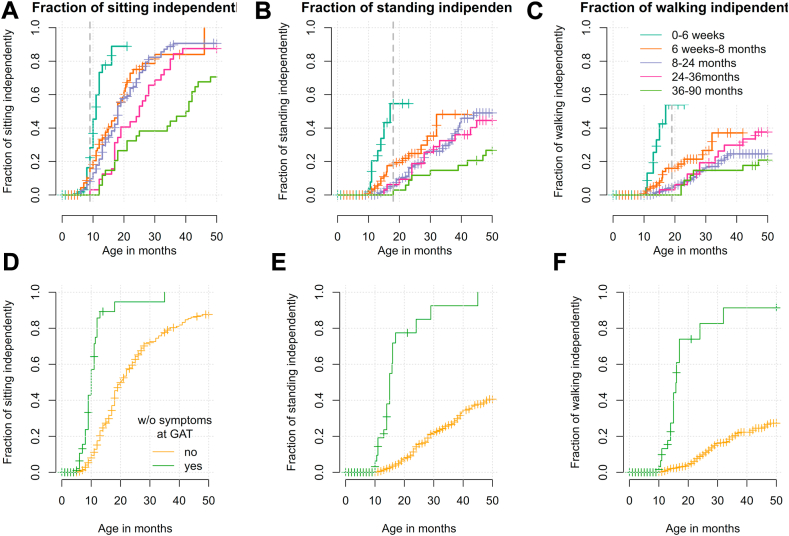
**Outcome of the motor milestone independent sitting, standing, and walking.** The probability to achieve the motor milestones of free sitting **(A)**, free standing **(B)**, and free walking **(C)** after GAT in the age categories ≤6 weeks (turquoise), 6 weeks to 8 months (orange), 8–24 months (purple), 24–36 months (pink), and 36–90 months (green). Vertical lines depict censored observations when milestone was not reached at the last recorded visit. Vertical dashed lines mark the 99th percentile for normal development according to the World Health Organization Multicentre Growth Reference Study [WHO-MGRS^12^]. Note that for >30 months follow-up, only few measurements are available. The graphs D, E and F show a Kaplan Meyer analysis of patients reaching the milestone sitting **(D)**, standing **(E)** and walking **(F)** depending on still being presymptomatic at time of GAT. Patients without symptoms at GAT (green) reach the milestones of **(D)** sitting, **(E)** standing and **(F)** walking significantly earlier and more often than patients with symptoms at GAT (p < 0.0001).

Milestone data was available in all 343 children. In general, the probability of achieving the milestones independent sitting, standing, and walking decreased significantly the older children were at GAT (Cox-model: sitting p < 0.001, standing p < 0.001, walking p = 0.003, Fig. 3A, B and C). *SMN2* copy number (Cox-model: sitting: p = 0.0154, standing: p < 0.001, and walking: p = 0.002) and manifestation of the disease (Cox-model: presymptomatic vs. symptomatic; sitting, standing, and walking p < 0.001) had significant influence on the achievement of new milestones (Fig. 3D, E and F; Supplementary Figs. S7 and S8).

Independent sitting was achieved in 217 patients (63.3%) at a median age of 18 months (range 5–66, IQR 12.0). Three patients lost the ability to sit after GAT. Children treated at ≤6 weeks, 6 weeks-8 months, 8–24 months, 24–36 and > 36 months reached free sitting at a median age of 11, 18, 18, 25 and 40.5 months, respectively (Supplementary Fig. S9). Out of children who were treated presymptomatically, free sitting was achieved by 43% within the normal window of achievement defined by the WHO and only by 8% who had developed symptoms before GAT (p < 0.0001; Fig. 3D, Supplementary Fig. S9).

Free standing was achieved in 82 patients (23.9%) at a median age of 23 months (range 10–59, IQR 16.8). No patient lost this ability after GAT. Patients with 3 *SMN2* copies were 3.9 times more likely to achieve free standing than those with 2 *SMN2* copies (Cox-model p < 0.001, Supplementary Fig. S7). In children who were treated presymptomatically, free standing was achieved by 78% within the age-appropriate time, but only by 5% of those treated symptomatically (p < 0.0001; Fig. 3E).

Free walking was achieved in 59 patients (17.2%) at a median age of 23 months (range 10–64, IQR 14.1). One patient lost this ability after GAT. Patients with 3 *SMN2* copies were 4.2 times more likely to achieve free walking than those with 2 *SMN2* copies (Cox-model, p < 0.001, Supplementary Fig. S7). In children who were treated presymptomatically, free walking was achieved by 74% within the age-appropriate time, but only by 5% of those treated symptomatically (p < 0.0001; Fig. 3F).

### Respiratory function and nutritional support

Data was available in all 343 children. Before GAT, 64 patients (18.7%) had either non-invasive (n = 60) or invasive (n = 4) ventilation support. The overall age patients received a ventilation support was at a median 15 months (range 0–94 months, IQR 27.5). During the observation period, 11/279 patients (3.9%) started on ventilation support after GAT, while 14/64 previously ventilated patients (21.9%) could be weaned off (McNemar’s Chi-squared test: p = 0.689).

The overall median age at which patients received a nutritional support was 19 months (range 0–106 months, IQR 30). Before GAT, 53 patients (15.5%) had nutritional support with a feeding tube or a percutaneous endoscopic gastrostomy. Of these, 23 (43.4%) were partially and 30 (56.6%) exclusively tube fed. Within the observation period, 7 patients (2.4%) of 290 patients without prior nutritional support had to be started on tube feeding while 3/53 patients (5.7%) who required nutritional support before GAT could be weaned off (McNemar’s Chi-squared test: p = 0.3428).

The probability of requiring either ventilation or nutritional support was significantly higher the older the patients were at the time of GAT (Cox model: p < 0.0001, Supplementary Fig. S10). Prior to treatment, 2/88 (2.3%) and 3/88 (3.4%) of the patients treated ≤6 weeks required ventilatory and nutritional support, respectively. After GAT, one additional patient needed ventilatory and nutritional support within the observation period. None of the pre-symptomatically treated patients needed respiratory or nutritional support in the observation period. The probability of requiring ventilation or nutritional support was lower in children with more *SMN2* copies: 72 of 207 patients (35%) with 2 *SMN2* copies required ventilation whereas only 6 of 136 patients (4%) with 3 *SMN2* copies needed respiratory support. 57 of 207 patients (28%) with 2 *SMN2* copies and 2 of 136 patients (1%) with 3 *SMN2* copies were tube fed.

### Safety data

**Fig. 4:**
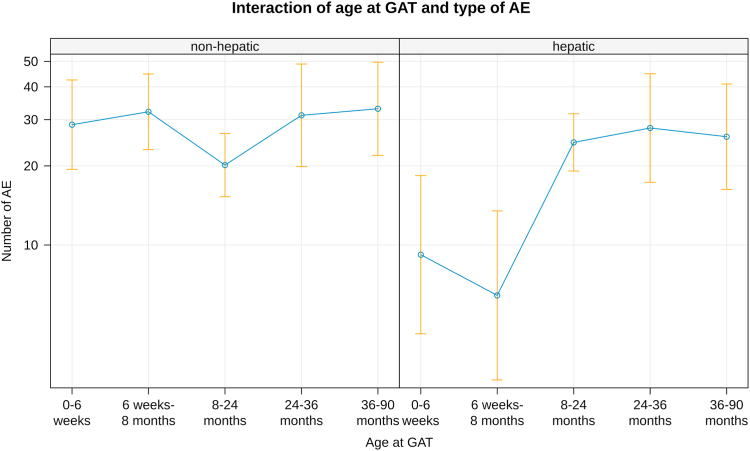
**Liver related adverse events.** Liver related adverse events were reported significantly higher in children >8 months of age at treatment whereas all other adverse events occurred age independent (p = 0.005 0–6 weeks at GAT; p < 0.0001 1–8 months).

263 adverse events were reported in 123 children (35.9%), including 40 events requiring hospitalization (Table 1). Liver related adverse events were reported in 93 children (27.1%) including 17 (5.0%) with severe hepatopathy. Thrombocytopenia was reported in 13 (3.8%) children with one child experiencing thrombotic microangiopathy^13^ and each one proteinuria and petechia. Elevated cardiac enzymes were reported in 17 (5.0%) children including one child with tachycardia and one with left ventricular hypertrophy. Fever was reported in 15 (4.4%) patients, vomiting in 18 (5.2%) patients, and erythema in 2 (0.6%) patients. There were significantly less liver-related adverse events in children <8 months of age at treatment (p = 0.005 0–6 weeks at GAT; p < 0.0001 1–8 months), whereas all other adverse events occurred age independent (Fig. 4).

Six patients (1.7%) died during the observation period, their deaths were rated as unrelated to GAT (Table 1, Supplementary Table S1).

## Discussion

In this study, we present data from 343 SMA patients who were treated with OA, including relevant patient subgroups such as 79 children who were clinically presymptomatic at the time of treatment. Stratification of our cohort by age at GAT confirmed that early treatment, in particular, ≤6 weeks-of-age, is the most important predictor for motor outcome and milestone achievement. Clinically presymptomatic children at first presentation (“presymptomatic”) are developing significantly better than symptomatic ones. A significant and robust effect of gene addition therapy can be reproduced in children up to 24 months with different statistical tests for CHOP INTEND, HFMSE as well as HINE-scores. In children older than 24 months motor outcome was stable over time but did not increase significantly across all outcome measures. The milestones of free sitting, standing, and walking were reached significantly earlier and more frequently the earlier the children were treated. That is, patients treated before the onset of clinical symptoms were able to achieve the motor milestones of independent sitting at a median age of 10 months and standing and walking at a median age of 15 months. In contrast, children who had SMA symptoms at the time of treatment achieved the ability to sit at a median age of 18.5 months but were unable to stand or walk within the observation period of this study. It is important to mention that although 134 patients were diagnosed soon after birth (117 via newborn screening, 15 via positive family history, 2 without further information available), only 79 of them were still classified as presymptomatic at time of GAT. These data are certainly relevant for counseling physicians and for parents to inform them about i) the pressing need for immediate start of treatment after diagnosis, ii) possible or probable motor impairments even after instant therapy of newborns and especially symptomatic patients at time of treatment and iii) the necessity and purpose of a bridging therapy if access to gene therapy is not immediately available. *SMN2* copy number was another significant influencing factor for motor outcome with a better outcome in patients with 3 compared to 2 *SMN2* copies. Thus, the patient’s own production of fully functional SMN protein might play an important role in complementing the effect of OA, and also in preventing early loss of motor neurons before OA administration. Previous treatment with *SMN2* splicing modifiers led to higher baseline motor scores, but did not significantly change disease trajectories.

Our findings are in line with results of recently published real-world-observations; however, their small sample sizes did not allow any stratification with regard to age at treatment, *SMN2* copy number, baseline motor and respiratory function: Waldrop et al.,^14^ Pane et al.,^15^ D'Silva et al.^16^ and Stettner et al.^17^ reported similar effects, with motor improvement depending on motor function at baseline, in 46 children after a mean follow-up of 33 months, in 42 children after 12-month follow-up, in 21 children after 15 months and in 9 patients after a 21-month follow-up respectively. Most recently, efficacy and safety of 97 patients with SMA type 1 and 2 presymptomatic patients from the UK was reported.^18^ As in our cohort, there was also a better response in terms of motor function in younger patients (<6 months) compared to children who were older at the time of therapy. In addition to the data from the UK cohort, our analysis identified onset of SMA symptoms and *SMN2* copy number as important influential factors and demonstrated that need for respiratory and feeding support remained stable after GAT. Real-world outcome from the Novartis-sponsored RESTORE-registry was collected for 168 patients treated with OA (without prior nusinersen or risdiplam) and showed favorable event-free-survival of OA treated patients compared with natural history data.^19^ In terms of motor outcome, data in a subset of patients (CHOP INTEND *n* = 41 patients, HFMSE *n* = 20, HINE *n* = 22 patients, motor milestones *n* = 45) showed also a trend towards better outcome in younger treated children. In addition to these data, we show that patients treated with nusinersen or risdiplam prior to OA start with higher motor scores, but this does not further influence the disease trajectories in terms of motor gain after OA.

Our results show that lower age at treatment and higher *SMN2* copy number positively influence not only motor development, but also respiratory outcome and swallowing. The need for ventilatory or nutritional support remained stable after treatment with OA in the overall cohort, also in the older and more affected patients. Some children could be weaned of either noninvasive ventilation (NIV) or tube feeding/PEG after GAT, a small percentage needed NIV or tube feeding despite OA treatment. However, neither the SMArtCARE registry nor the Swiss-Reg NMD collect data on other aspects of bulbar function, such as expressive speech or instrumental swallowing diagnostics. In the above-mentioned cohorts of the Italian, French, and Australian colleagues and the RESTORE registry, similar results were found for bulbar and respiratory function. In contrast, approximately 30% of symptomatic SMA type 1 patients treated with nusinersen showed a decline in bulbar and respiratory function at 38-month follow-up, while motor function improved.^11^ As reduced bioavailability in the upper spinal cord and motor nuclei may contribute to these findings,^20^ it is currently unknown whether systemic administration could overcome this problem in the long term.

With the inclusion of SMA in the Austrian and German newborn screening since June and October 2021, respectively, there has been the unique opportunity to treat most of these children with SMA identified through screening presymptomatically. Infants ≤6 weeks-of-age and without clinical signs of SMA at the time of GAT fared significantly better than those treated later and after onset of symptoms. Motor development was, however, less favorable in patients who had already developed SMA symptoms despite early treatment initiation, and a subset even required respiratory and nutritional support. Despite the same age cut-off at GAT, the SPRINT-study is therefore not comparable with the real-world-situation as only infants with neither clinical nor electrophysiological signs were included.^21^^,^^22^ In contrast, our large cohort of 88 infants treated in the neonatal period represents the broad range of newborns currently treated in the real world, including those with prenatal or early postnatal onset of SMA symptoms. While in the SPR1NT study 79% newborns with 2 *SMN2* copies and 100% of those with 3 *SMN2* copies reached the milestone of free sitting within the normal developmental window, this was only the case in 28% of the newborns in our cohort and 1.5 times more likely in those with 3 vs. 2 *SMN2* copies. Measurement of compound muscle action potential (CMAP) as an electrophysiological and earlier marker of disease onset than clinical symptoms is not routinely used in daily practice and therefore not included in this study. Thus, our real-world cohort may have included patients without clinical symptoms but with subclinical disease manifestations, this could explain the discrepant data. It supports the theory that high amounts of functional SMN-protein for synaptogenesis are needed even before birth, from about 32 weeks of gestational age.^23^ Thus, treatment initiation immediately after birth might still be too late for the achievement of normal motor development, especially in high-risk constellations such as clinical symptoms at first examination and/or 2 *SMN2* copies. Therefore, the therapeutic window between pathologic newborn screening, genetic confirmation of SMA, and initiation of treatment should be as streamlined as possible to avoid delays in treatment.

In terms of safety, OA was generally well tolerated in our cohort. There were no new, previously unknown safety alerts that are not yet reflected in the current version of the Summary of Product Characteristics (SmPC) of OA. Six children died within the observation period, all of them judged as unrelated to OA. Serious side effects occurred as severe hepatopathy (n = 17) and thrombotic microangiopathy (n = 1), all of them manageable with escalating immunosuppressive drug regimens. Most important finding in terms of safety in this cohort is the increased risk for hepatopathy with age in relationship to all other non-hepatic adverse events. While the frequency of non-hepatic AE’s remained stable across all age and weight ranges, the frequency of liver-associated AEs increased significantly in children older than 8 months. These data confirm previous findings in the above mentioned real-world-observations.

In conclusion, our study includes the so far largest international cohort of SMA patients treated with OA. We were able to statistically analyze subgroups of patients for the first time. As expected, age at treatment and *SMN2* copy number were important influencing factors on motor and bulbar outcome while previous treatment with *SMN2* splicing modifiers did not change disease trajectories. Notably, if and to what extent symptoms of SMA are clinically present at time of treatment is the most important prognostic factor of motor development. Risk-benefit-ratio increases the older and heavier children are at time of OA treatment. Based on our data, parents can therefore be better informed about realistic outcome expectations and possible risk factors of treatment with OA. As there will be no head-to-head studies comparing the three currently approved SMA treatments in different patient groups, larger registry studies will help to give guidance on which treatment to choose for individual patients. In the future, with most patients hopefully identified through NBS and early treatment, our predictive models including *SMN2* copy and baseline clinical status should be extended to offer valid counseling for this patient population.

Prior to the approval of GAT, therapy-independent academic registries for SMA patients already existed in German-speaking countries providing the unique opportunity to analyze this comprehensive data collection of 343 SMA patients. In particular, our cohort of children older and heavier than those treated in the pivotal trials, of infants treated ≤6 weeks-of-age, of those treated presymptomatically and with nusinersen or risdiplam prior to OA, and the long observation period of up to 43 months provide new insights into the effectiveness and safety of treatment with OA. These subpopulations were all analyzed according to the same criteria, and, for the first time, a comprehensive statistical comparison was possible using a prespecified statistical analysis plan. Our dataset offers prediction models of expected motor outcome in relation to time at treatment with OA and allows comparison of disease trajectories depending on clinical manifestation of the disease (symptomatic vs. presymptomatic) and *SMN2* copy number.

As a limitation of this study a comprehensive analysis of patients with more than 24 months of follow-up was not possible due to the smaller number of patients. Despite a structured German consensus plan for the follow-up after GAT, some data points on outcome parameters were missing in the follow-up period beyond 24 months, reflecting the data collection in a registry study rather than a controlled clinical trial. Since randomized controlled trials are unlikely to be conducted in the future after therapies are approved, it is important to establish more standardized follow-up documentation, including verification of source data. In addition, data collection should be regularly adapted to the changing therapeutic landscape and new questions that arise in relation to the phenotype of the disease under treatment (e.g., assessment of cognitive development). To be able to better compare the outcome between symptomatic and pre-symptomatic patients in the future, an electrophysiological examination of all newborns at diagnosis would be desirable.

We recommend refining the assessment and severity grading of safety events in future real-world-observations as we saw a large heterogeneity in safety reporting across different treatment centers. We observed a trend towards a less frequent AE reporting in centers with bigger OA treatment experience. There was a large uncertainty among treating physicians regarding the definition of “Adverse Events” and “Serious-Adverse Events” as these are terms used in pivotal trials. Therefore, we suggest the terms Adverse Drug Reaction (ADR) and Adverse Events of Special Interest (AESI) for future post-marketing-collections as they are more precisely defined and practicable in post-marketing-settings. All possible ADRs and AESIs should furthermore be clearly defined, precisely categorized and graded in terms of severity using systematic terminologies like the Common Terminology Criteria for Adverse Events (CTCAE). Applying these homogenous definitions, terminologies and severity gradings across different registries will improve comparability of safety outcomes of different gene-therapy-medicinal-products in post-marketing data collections.

We have calculated 2-fold interactions in order to map any differences in the profile progression of the motor scores. However, this may result in the groups to be compared becoming relatively small, which should be seen as a potential limitation of our study. Number of adverse events were only analyzed with respect to GAT to avoid small group sizes when adding more predictors. Therefore, we cannot exclude that there are more potential risk factors others than GAT leading to adverse events. Although mortality is low, we cannot exclude that this pose a risk to some analysis, e.g., as a competing event for adverse events.

## Contributors

CW and LLB equally contributed as first authors to the manuscript. JJ and AZ equally contributed as last authors to the manuscript. CW, LLB and JJ wrote the first draft of the report. Major role in acquisition, compilation and interpretation of data, access and verification of data. AZ: Major role of compilation of data, access and verification of data. Review of statistical analysis, revision, final review and decision to submit the manuscript. SFG: Statistical analysis, design of figures, access and verification of data, and revision of manuscript. JF, AB, AH, SI, OS, GB, MvdH, RAH, KG, JK, AP, MFB, GS, US, BP, RT, VH, EW, MB, AK, AE, CK, GMS, SC, OH: Acquisition of data, revision of manuscript. All authors have seen and approved of the final text.

## Data sharing statement

All data included in this analysis are recorded in the SMArtCARE and SwissReg-NMD registries. Data can be obtained anonymized and aggregated upon request and approval by the SMArtCARE or SwissReg-NMD steering committees, respectively. The study protocol is published.^24^

## Declaration of interests

No author received financial support for the present manuscript.

CW received honoraria for presentations and/or travel support from Novartis, Roche and Biogen and participated on advisory boards for Novartis, Roche and Biogen. JF received honoraria from Novartis for presentations. AB received honoraria for presentations from Roche and Pfizer and attend advisory boards for Roche and Pfizer. SI received honoraria for presentations and advisory board meetings from Novartis and Roche. OS received honoraria from Biogen, support for attending meetings from Novartis and participated on an advisory board for Novartis. GB received honoraria for advisory boards and/or presentations from Novartis, Roche and Biogen and support for attending meetings from Novartis. MvdH received grants from Deutsche Gesellschaft für Muskelkranke and Innovationsfond (INTEGRATE ATMP and KoCoN), honoraria from Pfizer, Biogen and PTC Therapeutics, and participated on advisory boards for Roche, Sarepta, Novartis, and Pfizer. RAH received consulting fees from Biogen and honoraria for presentations from Novartis, and participated on advisory boards by Novartis and Roche. KG participated on an industry symposium and an advisory board by Novartis. JK received funding for clinical research from Biogen, Novartis, Roche, ScholarRock and Biohaven, consulting fees from Biogen, Novartis and Roche, payment for educational activities from Biogen, Novartis and Roche and attended a data safety monitoring board for Biogen. AP received research funding form Roche, Novartis and Biogen. MFB received consulting fees for advisory boards from Roche, Novartis and Biogen and payment for presentations from Novartis and Biogen. GS received honoraria for a case report and participated on an advisory board for Novartis. US received honoraria for presentations from Biogen, Novartis and Roche and support for attending meetings from Roche and Novartis.

BP received honoraria for a presentation from Biogen and participated on advisory boards for Novartis and Biogen. RT received honoraria for presentations from Desitin, PTC and Roche and participated on advisory boards for PTC, Roche and Santhera. VH attended an adivisory board for Biogen. MB received compensation for advisory boards and speakers honoraria from Novartis, Biogen and Roche and compensation for travel costs to meetings from Roche. AK is clinical lead of the Swiss Reg NMD, attended advisory board meetings of Novartis and received honorarium for a presentation by Novartis. AE received honoraria and payment for expert testimony from Biogen, Roche and Novartis, support for attending meetings from Biogen and Roche and participated on an advisory board for Biogen, Roche and Novartis. GMS participated on advisory boards from Biogen, Novartis and Roche and is member of the steering board for Swiss-Reg-NMD. SC received payment for a presentation. JJ received compensation for advisory boards and funding for travel or speaker honoraria from Avexis/Novartis, Biogen, IFT, Roche, PTC, Pfizer and Sarepta Therapeutics. AZ received compensation for advisory boards and funding for travel or speaker honoraria from Avexis/Novartis, Biogen, ITF, Roche, Pfizer and Sarepta Therapeutics. LLB, AH, EW, CK, OH, and SFG indicated no potential conflicts of interest to disclose.
