# Supplementary material for: Efficacy and safety of gene therapy with onasemnogene abeparvovec in children with spinal muscular atrophy in the D-A-CH-region: a population-based observational study
Source: Lancet Reg Health Eur. 2024 Oct 7;47:101092. doi: 10.1016/j.lanepe.2024.101092 (PMC11492610; doi:10.1016/j.lanepe.2024.101092)
Supplement: Supplementary Figs. S1–S11 and Tables S1–S8 [file mmc1.pdf]

Supplementary Material

Real-world efficacy of onasemnogene abeparvovec in spinal muscular atrophy

Methods

Outcome measurements

In the event of a patient's death, the center completed an end of data collection form with the date and cause of the death. Information on the need for ventilation including the start and end dates as well as the type of ventilation (invasive versus non-invasive), frequency (daily use, occasional use or use only when ill), and time of use (nighttime, intermittent day time and continuous nighttime, continuous or intermittent during acute illness) was collected at each visit. To assess the nutritional status, use of a gastric or nasal feeding tube (supplemental or exclusive) was documented with start and end dates as well as any difficulties with swallowing or chewing.
Motor function was assessed by centrally trained physiotherapists. The appropriate motor function assessment tool was selected according to age and motor skills. The Children's Hospital of Philadelphia Infant Test of Neuromuscular Disorders (CHOP INTEND; maximum score 64) was administered to all children <2 years of age as well as in those >2 years without ability to sit (non-sitters). The Hammersmith Functional Motor Scale-Expanded (HFMSE; maximum score 66) was additionally assessed in patients >2 years of age and when the CHOP INTEND score was between 50 and 60. If the CHOP INTEND score was greater than 60, assessment was switched to the HFMSE in all patients. Revised Upper Limb Module (RULM; maximum score 37) was recommended in patients >2 years with ability to sit in wheelchair. The 6-minute walk test (6MWT) was performed in patients >3 years of age and able to walk independently. Because repeated 6MWT was available for only seven patients at time of data cut, this test was not included in the statistical analysis.
Motor milestones were assessed using section 2 of the Hammersmith Infant Neurological Examination (HINE) which evaluates a set of eight motor milestones: head control, sitting, voluntary grasp, ability to kick, rolling, crawling or bottom shuffling, standing and walking. Special attention was paid to the milestones of sitting, standing and walking. These milestones were counted as achieved, if either stable sitting, independent standing or independent walking were possible.

Figures

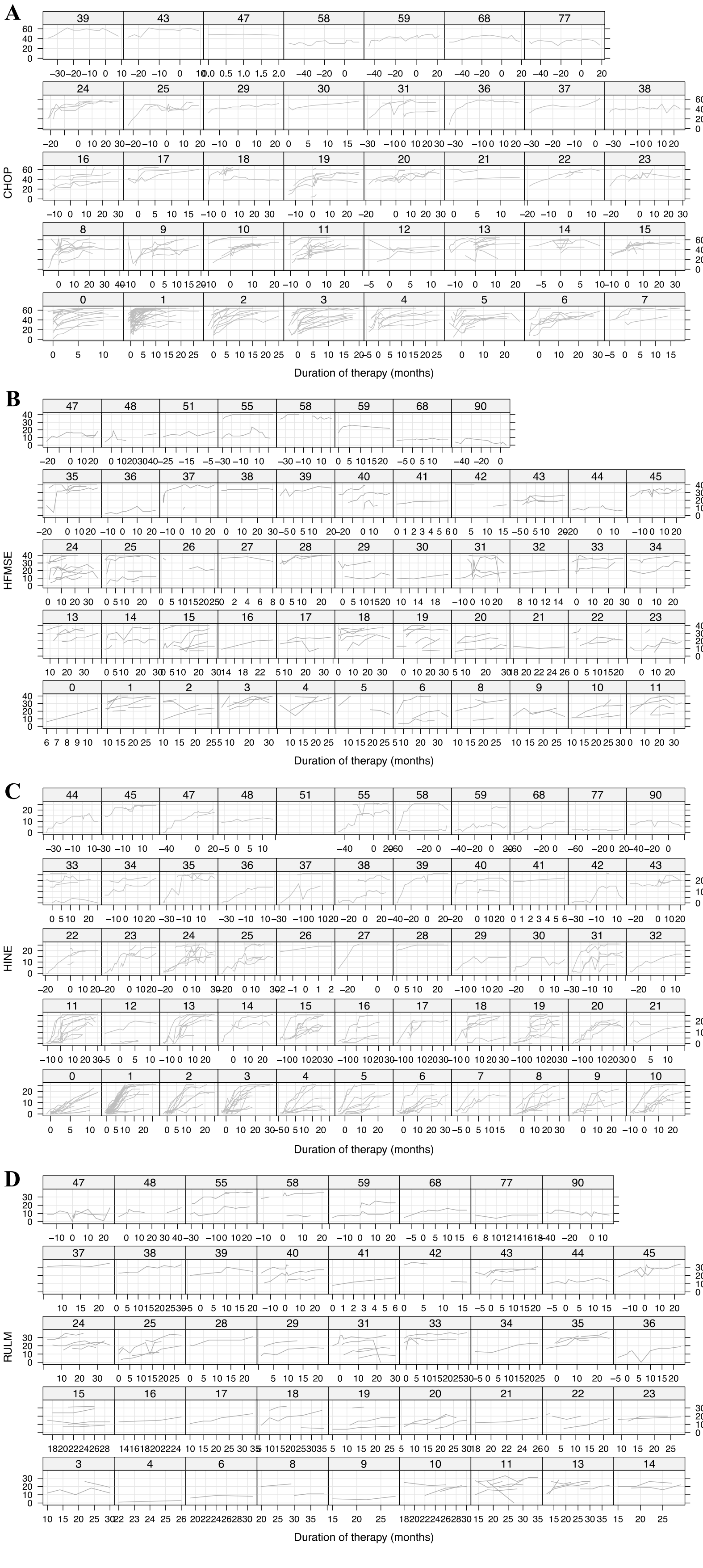

Supplementary Figure 1: Individual CHOP INTEND, HFMSE, HINE and RULM courses. Shown are the individual courses over the oberservation period in months of **(A) CHOP INTEND, (B) HFMSE, (C) HINE and (D) RULM courses.** Each panel depicts an age cohort in months at the time of GAT.

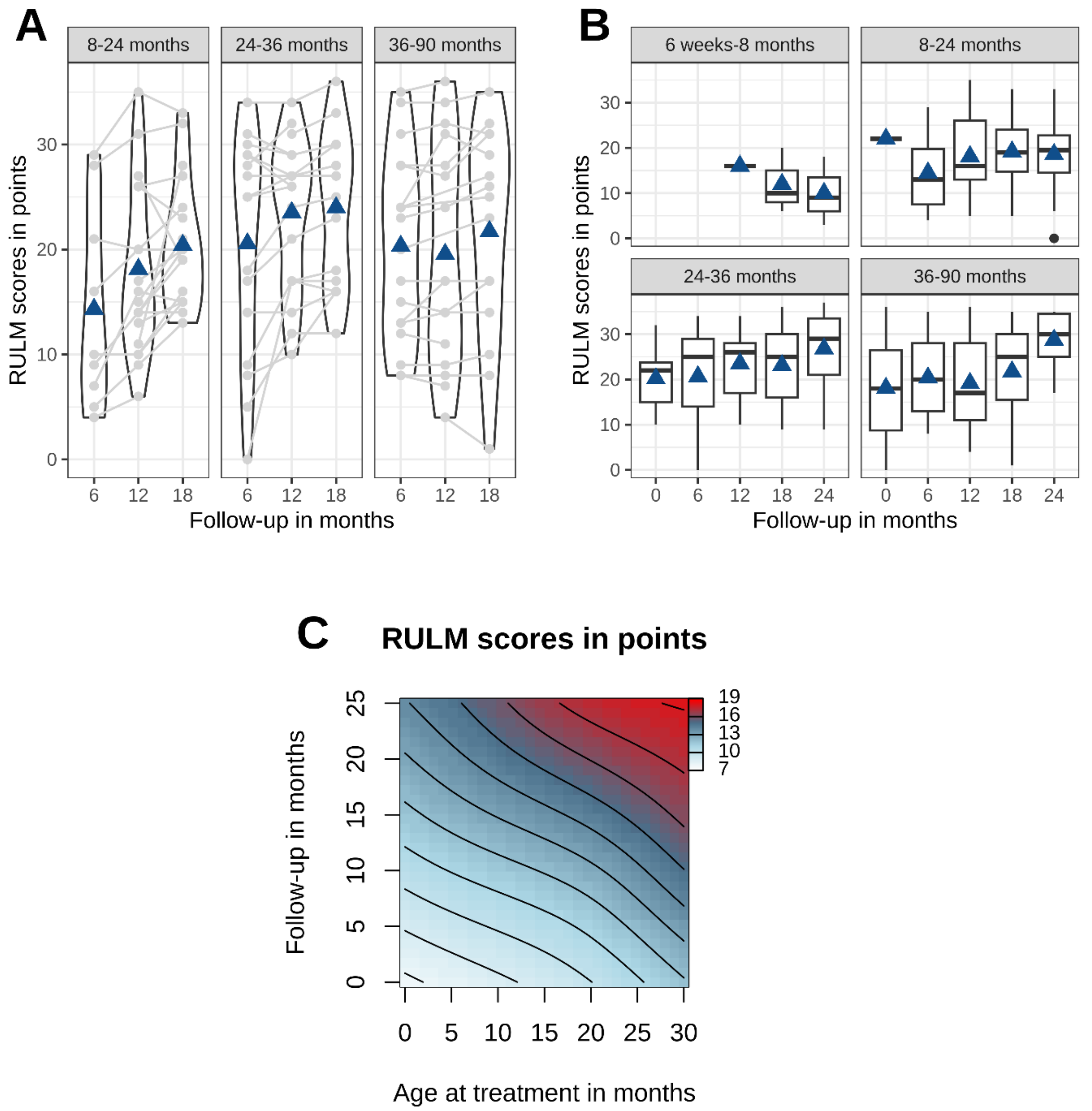

Supplementary Figure 2: **RULM scores before and after GAT.** RULM scores also increased 6 and 12 months after GAT; however, due to small sample size and test, no statistical analysis was performed. **(A)** Light gray lines depict individual courses, width of figures the number of patients represented and triangles mean values.

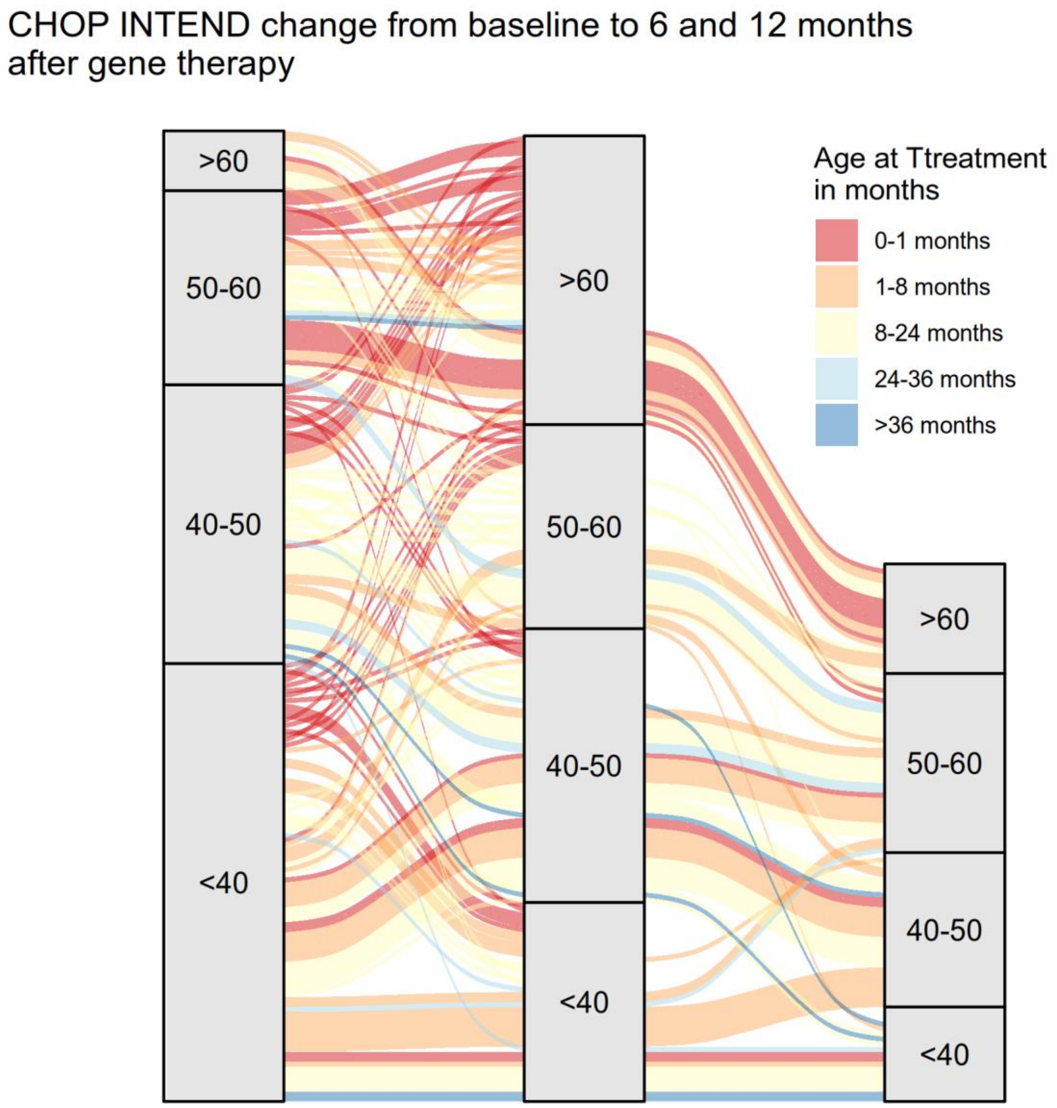

Supplementary Figure 3: Sankey diagram of CHOP INTEND scores at baseline, 6 months and 12 months after treatment. Shown are the individual courses of all patients with more than 2 CHOP INTEND score measurements at baseline, 6 months and 12 months after GAT. The columns represent CHOP INTEND categories < 40 between 40-50, between 50-60 and > 60 CHOP INTEND points.

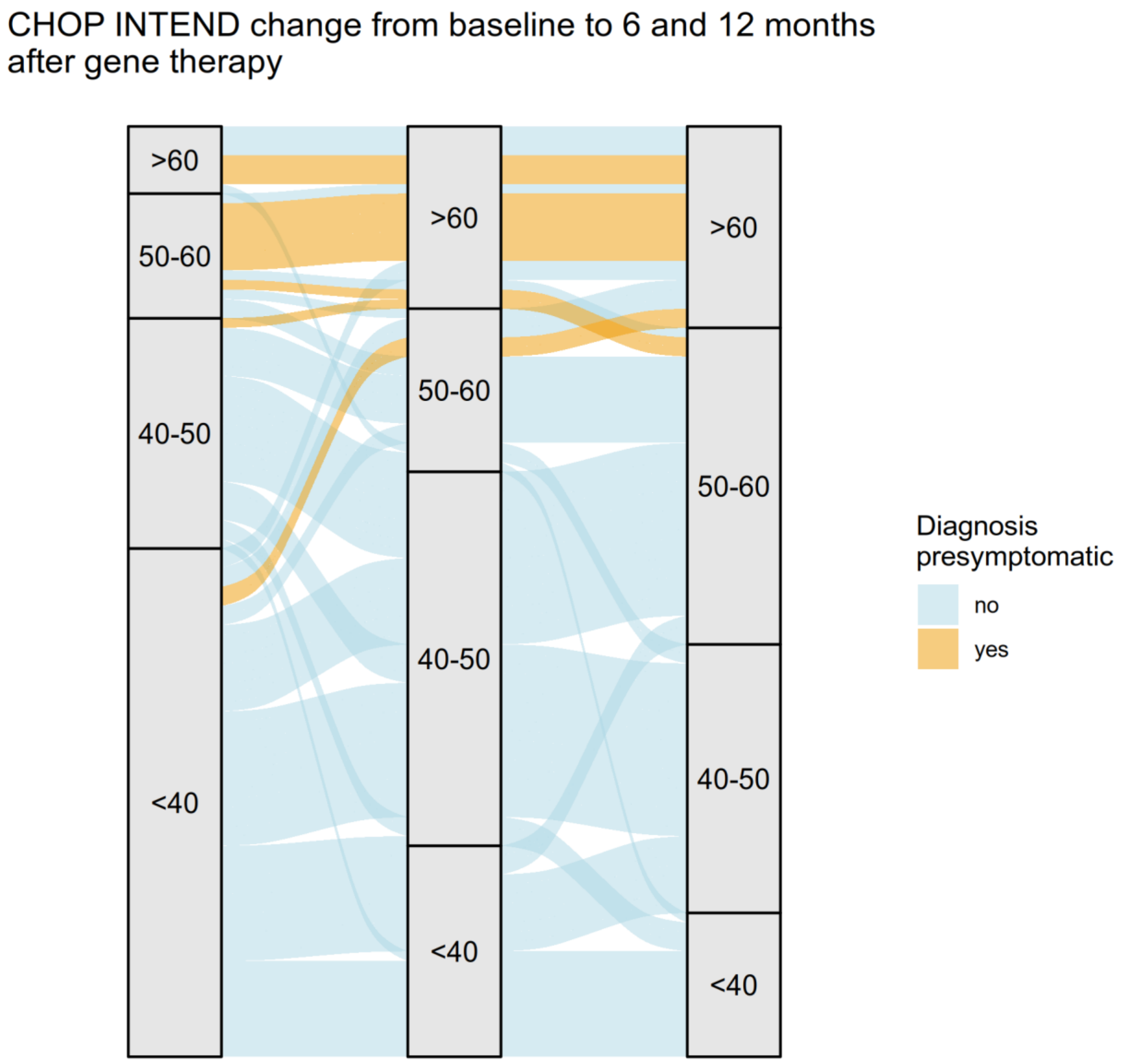

Supplementary Figure 4: Sankey diagram of CHOP INTEND scores treated pre-symptomatically and symptomatically at baseline, 6 months and 12 months after treatment. Shown are the individual courses of all patients identified with newborn screening with more than 2 CHOP INTEND score measurements at baseline, 6 months and 12 months after GAT. The columns represent CHOP INTEND categories < 40 between 40-50, between 50-60 and > 60 CHOP INTEND points.

.

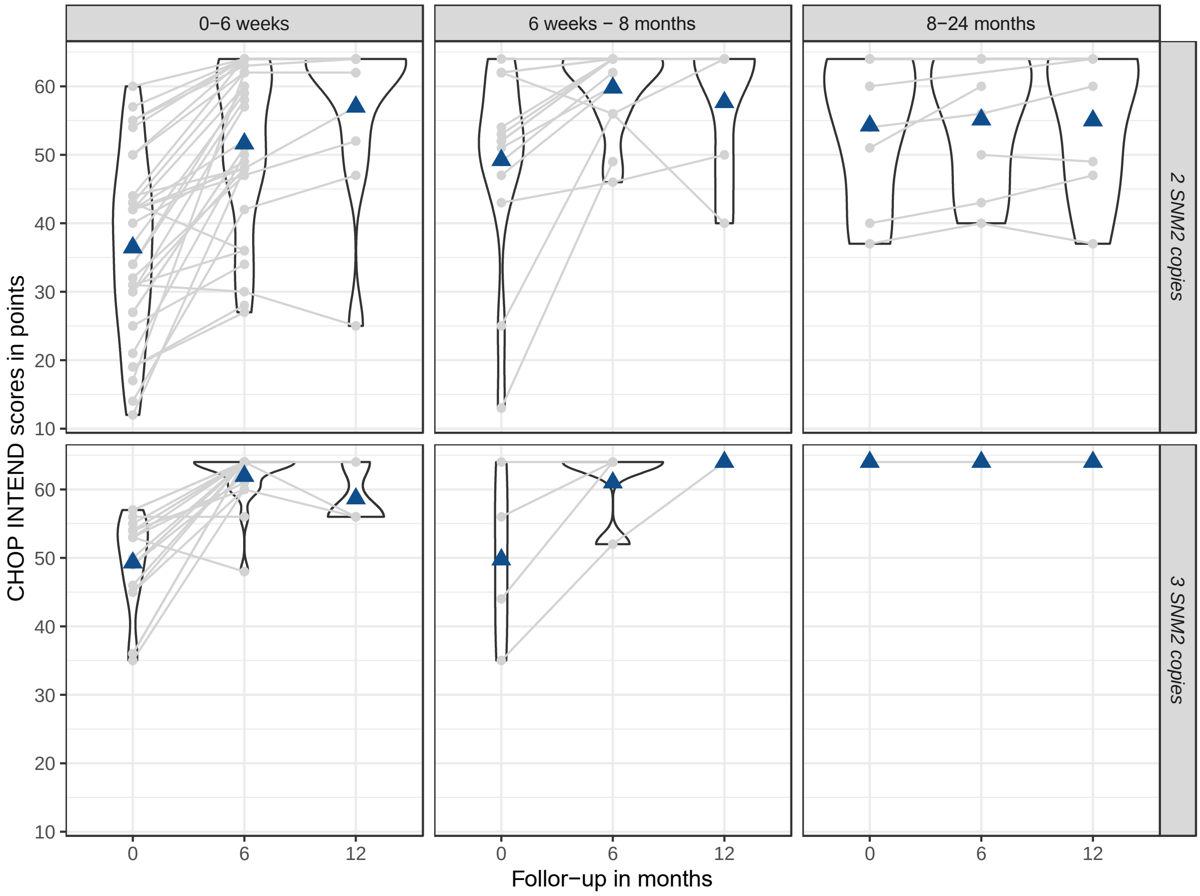

Supplementary Figure 5: Violinplot of patients identified with newborn screening. Shown are the CHOP INTEND scores at baseline (0) 6 and 12 months after GAT. The cohort is divided the different age-groups as well as 2 *SMN2* copy numbers (upper panels) and 3 *SMN2* copy numbers (lower panel). Patients identified with newborn screening with 3 *SMN2* copies. In patients > 8 months at GAT all but one patient received nusinersen before GAT.

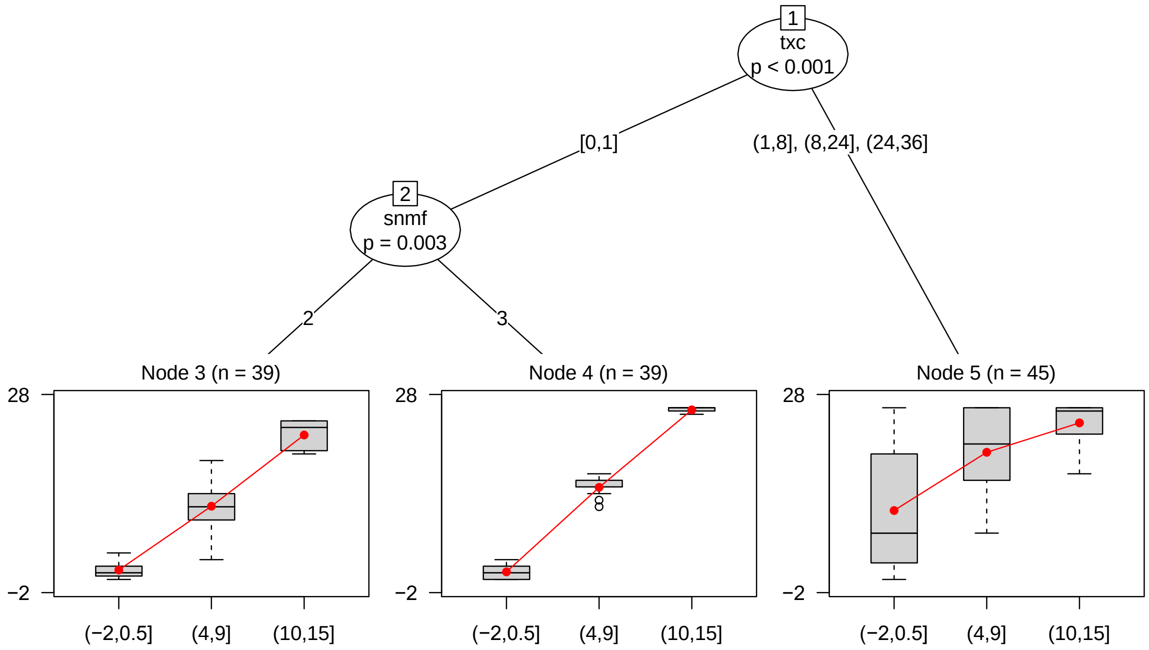

Supplementary Figure 6: Tree diagram of HINE scores in points of presymptomatic patients. Shown are boxplots of the HINE scores in points of pre-symptomatic patients in Note 4 to 7. Patients with a treatment in the age groups 0-6 weeks and 6 weeks to 8 months have a significantly higher HINE scores increase than those > 8 months (txc=age at treatment in months) in 1. Patients with a symptomatic diagnosis between 0-6 weeks have a significantly higher HINE scores increase than those between 6 weeks and 8 months in 2. Patients with a treatment between 0-6 weeks and with an *SMN2* copy number of 3 have a significantly higher HINE scores increase than those with 2 (snmf=*SMN2* copy number) in 3.

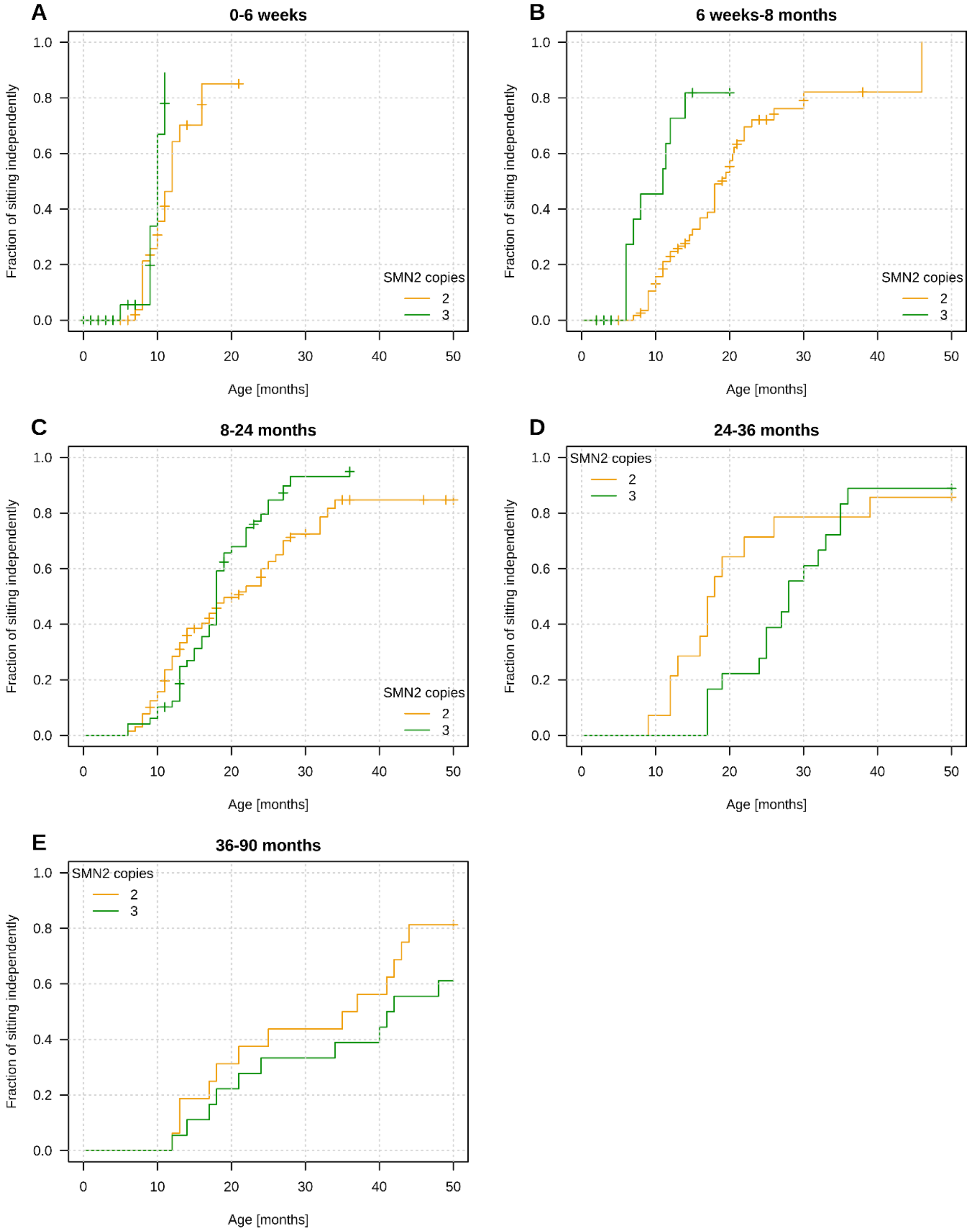

Supplementary Figure 7: Kaplan Meyer graph of patients reaching the milestone sitting, standing and walking depending on age groups at time of GAT and the *SMN2* copy number. The graphs show a Kaplan Meyer analysis of patients with 2 (green) and three (orange) *SMN2* copies in the different age groups **(A)** ≤6 weeks and **(B)** 6 weeks-8 months, **(C)** 8-24 months, **(D)** 24-36 months and **(E)** >36 months.

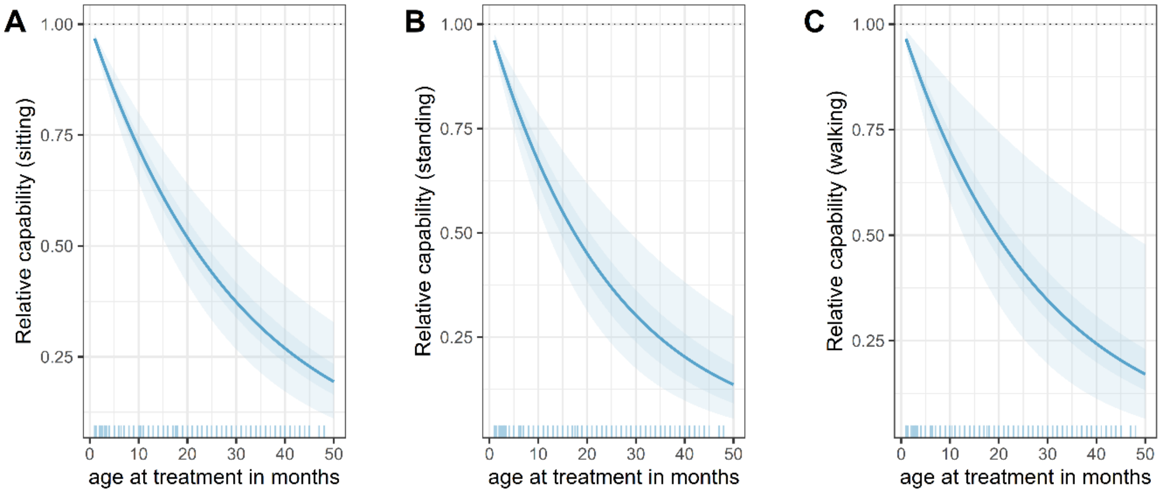

Supplementary Figure 8: Relative capacity of reaching the milestone (D) sitting, (E) standing and (F) walking estimated from Cox proportional hazard regression. The graphs show in a COX-model, that the older the patients are at GAT the less patients gain the milestone sitting, walking and standing (p<0·001).

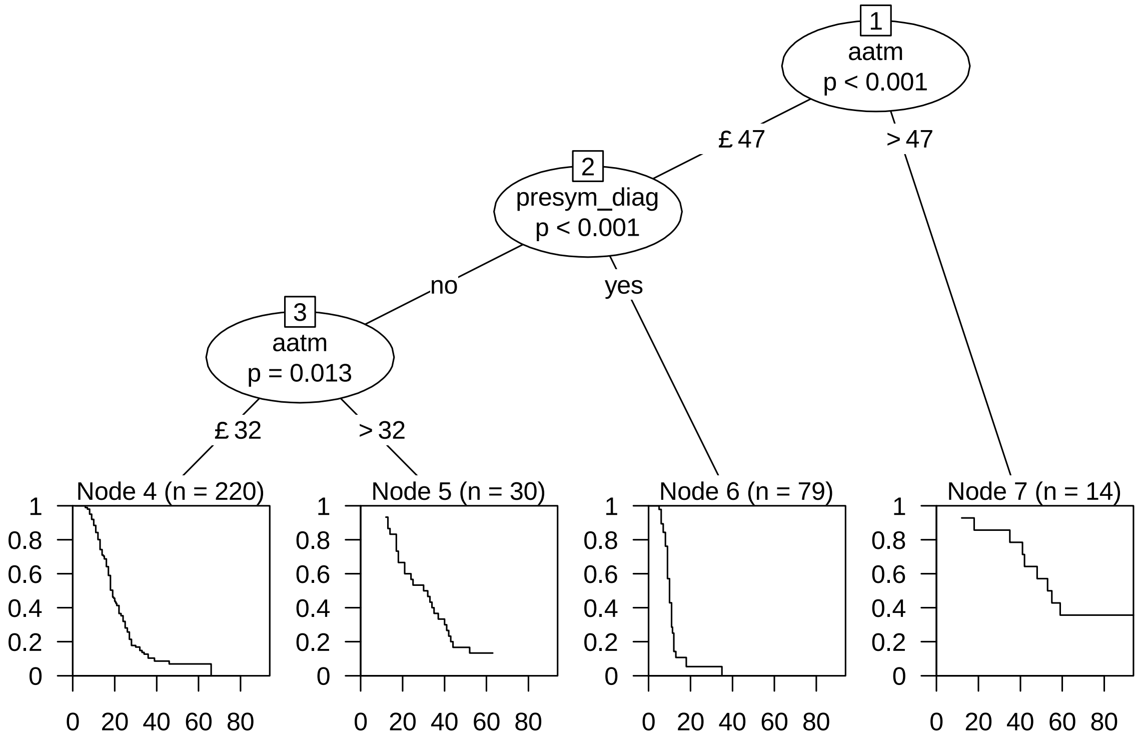

Supplementary Figure 9: Tree of patients reaching the milestone sitting. Shown are courses of patients reaching the milestone “sitting” in Note 3 to 7. Patients with a treatment > 47 months are significantly less frequently reaching the milestone (aatm=age at treatment in months) in 1. Patients with a symptomatic diagnosis significantly less frequently reaching the milestone (presym_diag=presympatomatic diagnosis) in 2. Patients with a treatment > 1 months are significantly less frequently reaching the milestone (aatm=age at treatment in months) in 3

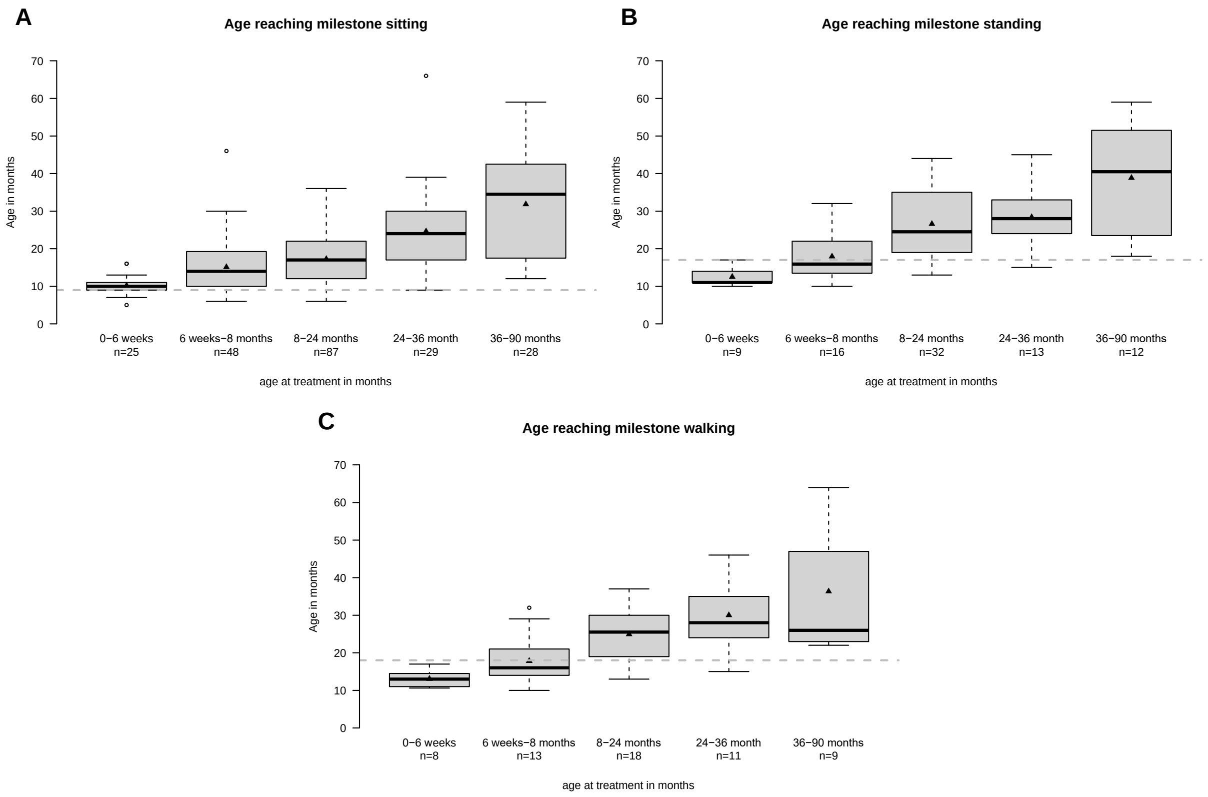

Supplementary Figure 10: Boxplot of median age of the milestones sitting, standing and walking. Shown are the boxplots of the age patients achieved the milestones of sitting (A), standing (B) and waling (C) devided in age-groups. The older the patients were at the time of GAT, the older they gained the milestone. The dotted line depicts the maximum normal range children should learn these milestones.

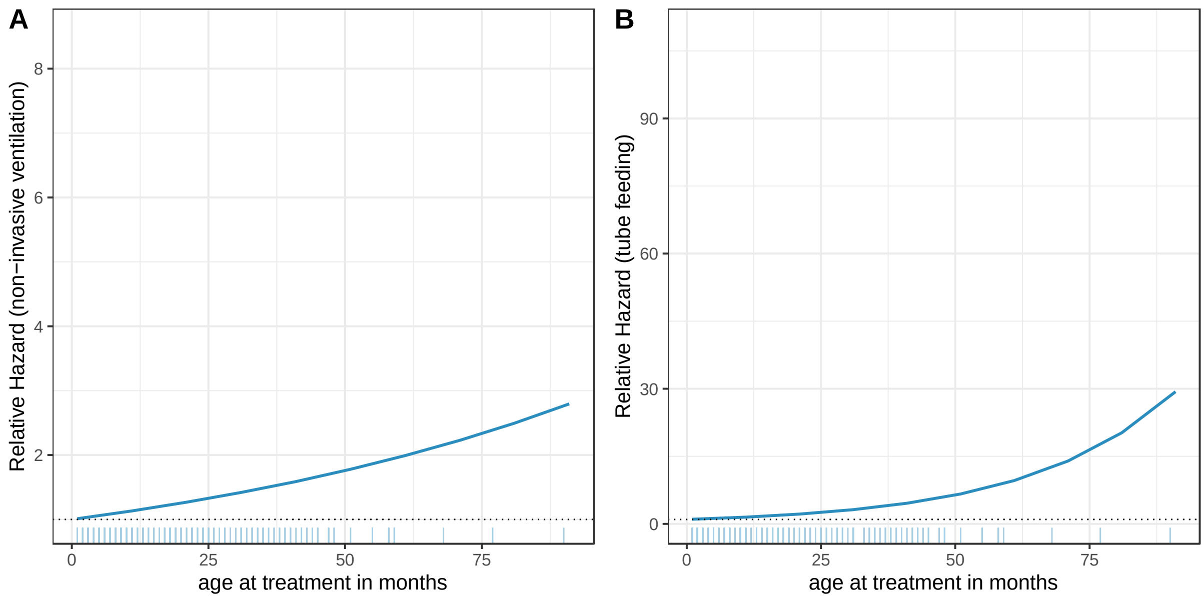

Supplementary Figure 11: Relative capacity of reaching ventilation and tube feeding estimated from Cox proportional hazard regression. Shown is the relative risk for respiratory support **(A)** and nutritional support **(B)** increases with higher age at treatment (p<0·0001).

Tables

P	gender	Age at infusion, months	Time death, months	Distance to infusion, months	reason
29003	F	9	12	3	Pneumonia/respiratory failure
44848	F	13	31	18	Cardiac arrest
49964	M	11	15	4	Aspiration/ cerebral hypoxia, termination of life support measures
62550	M	16	43	27	Respiratory infect, sudden death
99143	M	1	5	4	Respiratory insufficiency,<br>additional hypoplastic aorta, Deletion MIB1-Gen, duplikation in the PAR1 Region x-p22.23

Supplementary Table 1: Patient characteristics of lethal causes.

Influencing factors	Timepoint (months)	n	Mean±SD (range)	Mean change ±SD (range) t0-t6	Mean change ±SD (range) t0-t12
**Age**					
≤6 weeks	0	74	40·6±13·7 (2-60)	n=55<br>14·6±10·2 (-7-45)	n=16<br>14·0±12·7 (-6-45)
	6	55	54·8±11·5 (27-64)		
	12	16	55·4±11·4 (25-64)		
	18	6	56·2±11·7 (35-64)		
	24	2	61·0±4·2 (58-64)		
	>27	-	-		
6 weeks - 8 months	-6	21	29·1±16·7 (5-62)	n=52<br>11·9±9·0 (-6-37)	n=39<br>17·1±13·5 (-22-47)
	0	60	36·1±16·4 (9-64)		
	6	55	47·5±12·7 (17-64)		
	12	40	48·9±9·8 (25-64)		
	18	27	50·4±9·1 (31-64)		
	24	12	50·6±5·4 (40-58)		
	>27	4	48·8±7·2 (41-58)		
8 - 24 months	-6	39	38·4±13·7 (11-62)	n=68<br>6·2±6·5 (-14-26)	n=38<br>8·9±7·6 (-11-29)
	0	81	42·0±12·0 (4-64)		
	6	73	49·3±9·3 (26-64)		
	12	41	49·2±9·5 (22-64)		
	18	21	48·3±6·8 (35-60)		
	24	13	49·0±8·2 (33-60)		
	>27	8	47·1±7·6 (36-56)		
24 – 36 months	-6	6	46·0±10·5 (28-58)	n=9<br>1·9±3·4 (-3-7)	n=6<br>4·5±6·8 (-2-16)
	0	9	44·9±8·7 (30-58)		
	6	9	46·8±9·4 (33-60)		
	12	6	50·5±6·0 (39-56)		
	18	6	48·7±8·0 (34-56)		
	24	3	46·7±10·1 (35-53)		
	>27	1	36·0		
>36 months	-6	7	47·6±7·6 (38-58)	n=5<br>2·8±3·0 (0-7)	n=4<br>-0·8±5·5 (-8-4)
	0	7	44·6±9·7 (30-56)		
	6	7	44·0±8·2 (37-61)		
	12	5	37·2±6·9 (32-49)		
	18	4	37·5±9·0 (27-48)		
	24	1	41·0		
	>27	-	-		
**Sex**					
Female	-6	36	37·8±13·9 (5-62)	n=98<br>10·2±10·3 (-14-45)	n=54<br>12·3±13 4(-22-47)
	0	125	41·4±13·4 (4-64)		
	6	104	51·4±10·0 (28-64)		
	12	58	51·1±11·1 (22-64)		
	18	26	52·2±9·6 (31-64)		
	24	9	49·5±9·6 (33-60)		
	>27	2	45·5±10·6 (38-53)		
Male	-6	37	36·7±16·2 (5-64)	n=91<br>9·6±8·0 (-8-36)	n=49<br>12·0±9·8 (-6-33)
	0	106	38·8±14·2 (2-64)		
	6	95	48·3±12·2 (17-64)		
	12	50	47·8±·8 7 (25-64)		
	18	38	47·2±8·2 (27-64)		
	24	22	50·0±6·9 (35-64)		
	>27	11	47·0±7·6 (36-58)		

**Presymptomatic**					
Yes	-6<br>0<br>6<br>12<br>18<br>24<br>>27	5<br>60<br>42<br>15<br>5<br>1<br>-	42·2±13·5 (28-56)<br>48·7±9·5 (25-64)<br>60·4±6·5 (34-64)<br>62·1±4·0 (52-64)<br>64·0±0·0 (64-64)<br>64·0<br>-	n=42<br>11·6±8·1 (-5-31)	n=15<br>11·0±10·3 (0-39)
No	-6<br>0<br>6<br>12<br>18<br>24<br>>27	68<br>171<br>157<br>93<br>59<br>30<br>13	36·9±15·2 (5-62)<br>37·2±13·9 (2-64)<br>47·1±10·5 (17-64)<br>47·5±9·4 (22-64)<br>48·0±8·3 (27-64)<br>49·4±7·2 (33-60)<br>46·8±7·6 (36-58)	n=147<br>9·4±9·5 (-14-45)	n=88<br>12·4±12·1 (-22-47)
***SMN2* copy number**					
Two	-6<br>0<br>6<br>12<br>18<br>24<br>>27	59<br>154<br>140<br>88<br>51<br>27<br>12	35·6±15·8 (5-62)<br>36·6±14·4 (2-64)<br>47·8±11·2 (17-64)<br>49·0±10·5 (22-64)<br>48·9±8·3 (31-64)<br>50·0±7·5 (33-64)<br>47·7±7·1 (36-58)	n=131<br>10·7±9·5 (-8-45)	n=83<br>13·0±12·5 (-22-47)
Three	-6<br>0<br>6<br>12<br>18<br>24<br>>27	14<br>77<br>59<br>20<br>13<br>4<br>1	43·6±9·1 (28-58)<br>47·3±9·1 (27-64)<br>55·0±9·5 (31-64)<br>52·0±8·4 (35-64)<br>50·8±11·9 (27-64)<br>49·0±9·3 (35-54)<br>36·0	n=58<br>8·2±8·3 (-14-28)	n=20<br>8·6±7·6 (-2-29)
**Pretreatment**					
no	-6<br>0<br>6<br>12<br>18<br>24<br>>27	2<br>126<br>102<br>48<br>28<br>10<br>2	19·5±20·5 (5-34)<br>37·8±14·0 (2-60)<br>50·0±12·4 (17-64)<br>49·8±11·1 (25-64)<br>49·7±10·3 (31-64)<br>50·3±9·4 (35-64)<br>37·0±1·4 (36-38)	n=101<br>12·6±10·2 (-14-45)	n=48<br>16·3±12·5 (0-47)
nusinersen	-6<br>0<br>6<br>12<br>18<br>24<br>>27	65<br>91<br>88<br>59<br>35<br>21<br>11	38·2±15·0 (5-64)<br>43·3±12·7 (13-64)<br>49·8±9·9 (26-64)<br>49·2±9·4 (22-64)<br>48·5±7·8 (27-60)<br>49·7±6·8 (33-60)<br>48·5±6·8 (36-58)	n=80<br>7·3±6·9 (-6-36)	n=54<br>8·6±9·9 (-22-34)
risdiplam	-6<br>0<br>6<br>12<br>18<br>24<br>>27	5<br>12<br>8<br>1<br>1<br>-<br>-	36·8±9·1 (27-49)<br>41·7±16·7 (4-64)<br>52·4±10·0 (34-64)<br>61·0<br>64·0<br>-<br>-	n=7<br>3·9±5·1 (-3-12)	n=1<br>2·0
nusinersen<br>+risdiplam	-6<br>0<br>6<br>12<br>18<br>24<br>>27	1<br>2<br>1<br>-<br>-<br>-<br>-	16·0<br>42·5±3·5 (40-45)<br>37·0<br>-<br>-<br>-<br>-	n=1<br>-8·0	-

Supplementary Table 2: Influencing factors sex, presymptomatic diagnosis at treatment and *SMN2* copy number on CHOP INTEND score. Data are scores depicted in mean±SD (range) unless otherwise specified. CHOP INTEND=Children's Hospital of Philadelphia Infant Test of Neuromuscular Disorders. SMN=survival motor neuron gene.

Influencing factors	Timepoint (months)	n	Mean+SD (range)	Mean change ±SD (range) t0-t6	Mean change ±SD (range) t0-t12
**Age**					
≤6 weeks	t0<br>t6<br>t12<br>t18<br>t24	-<br>2<br>6<br>5<br>3	-<br>14·5±12·0 (6-23)<br>30·8±8·5 (20-40)<br>30·2±8·0 (21-40)<br>34·0±7·9 (25-40)	-	-

	>t27	1	37·0		
6 weeks - 8 months	t-6<br>t0<br>t6<br>t12<br>t18<br>t24<br>>t27	-<br>-<br>7<br>13<br>14<br>11<br>7	-<br>-<br>21·0±8·5 (4-28)<br>25·7±11·7 (4-40)<br>26·5±10·5 (9-40)<br>23·4±12·4 (7-39)<br>26·9±11·5 (8-40)	-	-
8 - 24 months	t-6<br>t0<br>t6<br>t12<br>t18<br>t24<br>>t27	2<br>13<br>35<br>47<br>41<br>39<br>21	10·5±3·5 (8-13)<br>25·4±9·2 (9-36)<br>22·5±11·0 (5-40)<br>22·5±10·4 (6-40)<br>24·3±9·9 (7-40)<br>23·9±9·7 (7-40)<br>23·7±8·6 (10-37)	n=8<br>4·6±3·6 (0-9)	n=10<br>9·2±7·3 (-1-21)
24 – 36 months	t-6<br>t0<br>t6<br>t12<br>t18<br>t24<br>>t27	9<br>21<br>25<br>24<br>21<br>12<br>3	27·4±12·9 (4-40)<br>23·6±11·1 (2-36)<br>27·3±11·9 (6-40)<br>26·7±12·6 (5-40)<br>26·9±12·6 (5-40)<br>30·8±10·6 (7-40)<br>27·7±13·6 (12-36)	n=20<br>5·5±6·5 (-4-25)	n=16<br>6·1±5·1 (0-23)
>36 months	t-6<br>t0<br>t6<br>t12<br>t18<br>t24<br>>t27	14<br>20<br>20<br>16<br>13<br>7<br>2	20·6±12·8 (2-40)<br>24·0±12·3 (3-40)<br>24·0±11·7 (0-40)<br>23·5±12·9 (6-40)<br>25·6±10·8 (7-36)<br>31·3±8·6 (18-40)<br>23·5±14·8 (13-34)	n=17<br>1·5±4·5 (-4-11)	n=13<br>0·3±4·7 (-5-12)
**Sex**<br>Female	 -6<br>0<br>6<br>12<br>18<br>24<br>>27	 14<br>36<br>51<br>63<br>54<br>38<br>14	 23·4±13·6 (2-40)<br>25·0±10·9 (2-340)<br>25·4±11·7 (0-40)<br>26·3±11·4 (5-40)<br>26·2±10·9 (7-40)<br>27·1±10·1 (7-40)<br>27·1±10·1 (12-40)	 n=30<br>3·9±5·2 (-4-20)	 n=27<br>5·2±6·5 (-5-21)
Male	-6<br>0<br>6<br>12<br>18<br>24<br>>27	11<br>18<br>38<br>43<br>40<br>34<br>20	20·8±12·5 (4-40)<br>22·4±11·1 (5-35)<br>21·8±10·7 (4-40)<br>21·8±11·1 (4-40)<br>25·0±10·1 (5-40)<br>24·9±11·0 (7-40)<br>23·7±9·4 (8-37)	n=15<br>3·7±6·5 (-4-25)	n=12<br>4·3±6·8 (-5-23)
**Presymptomatic**<br>Yes	 -6<br>0<br>6<br>12<br>18<br>24<br>>27	 2<br>2<br>8<br>8<br>7<br>4<br>2	 37·0±4·2 (34-40)<br>34·5±2·1 (33-36)<br>27·4±10·0 (6-40)<br>32·6±9·2 (14-40)<br>38·7±2·0 (35-40)<br>38·8±2·5 (35-40)<br>37·5±3·5 (35-40)	 n=2<br>-0·5±5·0 (-4-3)	 n=1<br>4·0
No	-6<br>0<br>6<br>12<br>18<br>24<br>>27	23<br>52<br>81<br>98<br>87<br>68<br>32	21·0±12·6 (2-40)<br>23·8±11·0 (2-36)<br>23·5±11·4 (0-40)<br>23·8±11·4 (4-40)<br>24·6±10·2 (5-40)<br>25·4±10·3 (7-40)<br>24·3±9·4 (8-40)	n=43<br>4·0±5·6 (-4-25)	n=38<br>5·0±6·6 (-5-23)
***SMN2* copy number**<br>Two	 -6<br>0<br>6<br>12<br>18<br>24<br>>27	 12<br>16<br>38<br>54<br>52<br>39<br>23	 17·1±12·2 (4-40)<br>19·0±11·4 (2-36)<br>18·8±10·2 (4-40)<br>19·6±10·1 (4-40)<br>21·6±10·3 (5-40)<br>22·5±10·9 (7-40)<br>22·6±10·1 (8-36)	 n=14<br>2·5±3·5 (-4-9)	 n=10<br>4·0±8·3 (-5-21)
Three	-6<br>0	13<br>38	27·1±12·1 (2-40)<br>26·3±10·1 (3-36)	n=31<br>4·4±6·3 (-4-25)	n=29<br>5·3±5·9 (-5-23)

	6<br>12<br>18<br>24<br>>27	51<br>52<br>42<br>33<br>11	27·6±10·7 (0-40)<br>29·5±10·5 (6-40)<br>30·8±8·5 (13-40)<br>30·4±8·3 (17-40)<br>30·4±6·4 (17-36)		-
**Pretreatment**<br>no	-6<br>0<br>6<br>12<br>18<br>24<br>>27	-<br>17<br>26<br>32<br>27<br>20<br>4	-<br>27·8±8·3 (10-40)<br>28·2±11·1 (4-40)<br>29·0±10·7 (4-40)<br>28·2±9·8 (9-40)<br>27·8±10·4 (7-40)<br>30·0±11·4 (13-37)	n=15<br>6·0±5·4 (-2-20)	n=13<br>7·4±5·5 (-1-20)
nusinersen	-6<br>0<br>6<br>12<br>18<br>24<br>>27	24<br>36<br>61<br>73<br>67<br>52<br>30	22·7±13·1 (2-40)<br>22·8±11·6 (2-40)<br>22·7±11·2 (0-40)<br>22·4±11·3 (5-40)<br>24·7±10·7 (5-40)<br>25·5±10·56(7-40)<br>24·4±9·5 (8-40)	n=28<br>2·7±5·5(-2-25)	n=26<br>3·7±6·8 (-5-23)
risdiplam	-6<br>0<br>6<br>12<br>18<br>24<br>>27	1<br>1<br>2<br>1<br>-<br>-<br>-	13·0<br>9·0<br>23·5±2·1 (22-25)<br>28·0<br>-<br>-<br>-	-	-

Supplementary Table 3: Influencing factors sex, presymptomatic diagnosis at treatment and *SMN2* copy number on HFMSE score. Data are scores depicted in mean±SD (range) unless otherwise specified. SMN=survival motor neuron gene. HFMSE=Hammersmith Functional Motor Scale-Expanded.

Influencing factors	Timepoint (months)	n	Mean+SD (range)	Mean change ±SD (range) t0-t6	Mean change ±SD (range) t0-t12
**Age**<br>≤6 weeks	t0<br>t6<br>t12<br>t18<br>t24<br>>t27	-<br>-<br>-<br>-<br>-<br>-	-<br>-<br>-<br>-<br>-<br>-	-	-
6 weeks - 8 months	t-6<br>t0<br>t6<br>t12<br>t18<br>t24<br>>t27	-<br>-<br>-<br>-<br>-<br>-<br>-	-<br>-<br>-<br>-<br>-<br>-<br>-	-	-
8 - 24 months	t-6<br>t0<br>t6<br>t12<br>t18<br>t24<br>>t27	-<br>1<br>10<br>25<br>32<br>30<br>21	-<br>22·0<br>14·5±9·1 (4-29)<br>18·1±8·3 (5-35)<br>19·2±6·9 (5-33)<br>18·6±7·6 (0-33)<br>20·0±6·8 (5-31)	-	-
24 - 36months	t-6<br>t0<br>t6<br>t12<br>t18<br>t24<br>>t27	4<br>10<br>17<br>19<br>17<br>11<br>4	14·0±6·1 (6-20)<br>20·2±7·5 (10-32)<br>20·6±10·2 (0-34)<br>23·5±7·1 (10-34)<br>23·1±7·9 (9-36)<br>26·8±8·6 (9-37)<br>24·3±12·9 (8-36)	n=9<br>3·6±5·7 (-10-8)	n=8<br>5·9±4·9 (-3-14)
>36 months	t-6<br>t0<br>t6<br>t12<br>t18<br>t24	14<br>20<br>23<br>18<br>18<br>10	18·8±9·6 (6-31)<br>18·1±10·8 (0-36)<br>20·4±9·0 (8-35)<br>19·0±10·2 (4-36)<br>20·1±10·2 (1-35)<br>23·1±11·2 (3-35)	n=16<br>2·6±2·7 (-2-9)	n=11<br>2·2±2·6 (-2-7)

	>t27	7	17·6±8·5 (8-33)		
**Sex**					
Female	-6	11	18·6±8·9 (9-31)	n=15	n=12
	0	18	19·8±9·6 (0-36)	3·8±2·9 (-2-8)	4·6±4·3 (-2-14)
	6	24	21·5±9·0 (5-35)		
	12	33	21·9±8·3 (4-36)		
	18	33	21·0±7·8 (1-36)		
	24	26	21·6±9·3 (3-37)		
	>27	14	20·8±7·8 (5-36)		
Male	-6	7	16·3±9·6 (6-30)	n=10	n=7
	0	13	17·7±9·7 (7-33)	1·7±5·1 (-10-9)	2·3±3·5 (-3-7)
	6	26	17·2±9·7 (0-34)		
	12	29	17·9±8·9 (5-35)		
	18	34	19·8±8·6 (5-33)		
	24	25	20·9±9·0 (0-35)		
	>27	18	19·3±8·3 (8-33)		
**Presymptomatic**					
Yes	-6	-	-	-	-
	0	-	-		
	6	1	25·0		
	12	1	27·0		
	18	3	28·0±2·6 (10-31)		
	24	2	16·0±22·6 (0-32)		
	>27	1	19·0		
No	-6	18	17·7±9·0 (6-31)	n=25	n=19
	0	31	18·9±9·6 (0-36)	3·0±4·0 (-10-9)	3·7±4·1 (-3-14)
	6	49	19·2±9·6 (0-35)		
	12	61	19·9±8·8 (4-36)		
	18	64	20·1±8·2 (1-36)		
	24	49	21·5±8·6 (3-37)		
	>27	31	20·0±8·1 (5-36)		
**SMN2 copy number**					
Two	-6	9	14·6±7·1 (6-30)	n=7	n=5
	0	9	12·1±9·3 (0-33)	0·7±5·9 (-10-9)	3·4±3·9 (-2-7)
	6	20	14·5±9·1 (0-34)		
	12	25	14·9±6·5 (4-27)		
	18	31	16·5±6·9 (1-31)		
	24	24	17·4±7·1 (3-32)		
	>27	21	16·7±6·0 (5-26)		
Three	-6	9	20·9±9·9 (6-31)	n=18	n=14
	0	22	21·7±8·4 (6-36)	3·8±2·7 (-1-8)	3·9±4·3 (-3-14)
	6	30	22·5±8·6 (8-35)		
	12	37	23·5±8·4 (8-36)		
	18	36	23·8±7·7 (8-36)		
	24	27	24·7±9·4 (0-37)		
	>27	11	26·3±7·7 (8-36)		
**Pretreatment**					
no	-6	-	-	n=6	n=3
	0	7	20·1±7·3 (10-33)	5·0±2·6 (1-8)	9·7±4·0 (6-14)
	6	8	23·8±5·9 (16-34)		
	12	12	23·9±6·7 (1-32)		
	18	13	22·4±6·5 (9-32)		
	24	11	18·3±10·7 (3-34)		
	27	6	17·8±10·3 (5-33)		
nusinersen	-6	18	17·7±9·0 (6-31)	n=19	n=16
	0	24	18·5±10·3 (0-36)	2·3±4·1 (-10-9)	2·6±3·1 (-3-7)
	6	42	18·5±9·9 (0-35)		
	12	50	19·1±9·0 (4-36)		
	18	54	20·0±8·5 (1-36)		
	24	40	22·1±8·5 (0-37)		
	>27	26	20·5±7·5 (8-36)		

Supplementary Table 4 Influencing factors sex, SMA type and SMN2 copy number on RULM score. Data are scores depicted in mean±SD (range) unless otherwise specified. SMN=survival motor neuron gene. RULM= Revised Upper Limb Module.

Influencing factors	Timepoint (months)	n	Mean+SD (range)	Mean change ±SD (range) t0-t6	Mean change ±SD (range) t0-t12
**Age**					
≤6 weeks	t0	62	1·1±1·1 (0-4)	n=46	n=17
	t6	46	9·7±5·1 (1-18)	8·6±4·8 (1-17)	18·6±7·0 (1-25)
	t12	17	19·6±7·4 (2-26)		
	t18	7	20·0±7·8 (4-26)		

	t24<br>>t27	3<br>-	23·0±5·2 (17-26)<br>-		
6 weeks - 8 months	t-6<br>t0<br>t6<br>t12<br>t18<br>t24<br>>t27	21<br>61<br>52<br>41<br>32<br>14<br>7	1·5±1·5 (0-5)<br>2·9±3·8 (0-16)<br>9·2±6·4 (0-26)<br>13·8±7·7 (1-26)<br>16·0±7·1 (3-26)<br>16·4±6·9 (3-26)<br>19·9±7·6 (8-26)	n=52<br>6·3±4·7 (-2-17)	n=41<br>11·1±6·4 (0-26)
8 - 24 months	t-6<br>t0<br>t6<br>t12<br>t18<br>t24<br>>t27	47<br>97<br>86<br>70<br>53<br>35<br>12	6·3±5·9 (0-24)<br>10·8±7·0 (0-25)<br>15·3±6·9 (2-26)<br>17·4±6·8 (4-26)<br>18·4±6·4 (2-26)<br>19·6±5·5 (5-26)<br>15·0±7·1 (5-26)	n=85<br>4·1±3·7 (-7-16)	n=69<br>6·0±4·9 (-6-18)
24 - 36months	-6<br>0<br>6<br>12<br>18<br>24<br>>27	15<br>28<br>26<br>24<br>19<br>12<br>2	17·6±6·1 (10-26)<br>17·5±7·2 (0-26)<br>18·3±7·7 (0-26)<br>19·2±6·4 (4-26)<br>18·2±6·9 (3-26)<br>19·8±7·7 (0-26)<br>20·0±8·5 (14-26)	n=26<br>0·7±3·0 (-7-6)	n=24<br>1·8±3·3 (-6-8)
>36 months	-6<br>0<br>6<br>12<br>18<br>24<br>>27	20<br>29<br>26<br>22<br>15<br>5<br>1	15·6±7·3 (2-26)<br>16·2±7·1 (2-26)<br>17·3±7·4 (2-26)<br>16·1±8·0 (3-26)<br>18·4±7·7 (3-26)<br>19·8±6·6 (9-26)<br>21·0	n=25<br>1·6±2·0 (-2-7)	n=21<br>1·0±3·6 (-6-8)
**Sex**<br>Female	-6<br>0<br>6<br>12<br>18<br>24<br>>27	53<br>153<br>129<br>97<br>61<br>28<br>8	8·9±8·5 (0-26)<br>8·5±8·4 (0-26)<br>14·6±6·9 (1-26)<br>18·6±6·6 (2-26)<br>19·7±6·1 (2-26)<br>20·4±6·1 (3-26)<br>18·0±8·7 (5-26)	n=129<br>5·5±5·1 (-7-17)	n=97<br>8·7±8·1 (-6-26)
Male	-6<br>0<br>6<br>12<br>18<br>24<br>>27	50<br>124<br>107<br>77<br>65<br>41<br>14	8·6±7·6 (0-26)<br>7·6±7·8 (0-26)<br>12·0±7·8 (0-26)<br>14·7±7·8 (1-26)<br>16·1±7·1 (3-26)<br>18·3±6·4 (0-26)<br>16·9±6·6 (5-26)	n=105<br>4·0±4·1 (-6-16)	n=75<br>5·3±5·7 (-6-23)
**Presymptomatic**<br>Yes	-6<br>0<br>6<br>12<br>18<br>24<br>>27	11<br>60<br>43<br>20<br>11<br>4<br>2	9·8±10·9 (0-26)<br>4·2±6·9 (0-26)<br>15·0±5·7 (3-26)<br>23·4±3·0 (16-26)<br>25·2±1·3 (23-26)<br>25·5±1·0 (24-26)<br>26·0±0·0 (26-26)	n=43<br>10·1±4·7 (-1-17)	n=20<br>16·5±8·6 (0-26)
No	-6<br>0<br>6<br>12<br>18<br>24<br>>27	92<br>217<br>193<br>154<br>115<br>65<br>20	8·7±7·7 (0-26)<br>9·2±8·1 (0-26)<br>13·1±7·8 (0-26)<br>16·0±7·4 (1-26)<br>17·1±6·8 (2-26)<br>18·7±6·3 (0-26)<br>16·4±7·1 (5-26)	n=191<br>3·6±3·8 (-7-16)	n=152<br>6·0±6·2 (-6-21)
**SMN2 copy number**<br>Two	-6<br>0<br>6<br>12<br>18<br>24<br>>27	74<br>166<br>145<br>111<br>80<br>43<br>16	7·1±7·3 (0-26)<br>6·1±7·1 (0-26)<br>10·7±7·1 (0-26)<br>14·9±7·3 (1-26)<br>16·5±7·0 (2-26)<br>17·7±7·0 (0-26)<br>15·6±7·7 (5-26)	n=143<br>4·5±4·2 (-7-17)	n=109<br>7·8±7·2 (-6-23)
Three					

	-6	29	13·0±8·5 (1-26)	n=91	n=63
	0	111	11·3±8·7 (0-26)	5·3±5·4 (-7-17)	6·3±7·3 (-6-26)
	6	91	17·27±5·9 (2-26)		
	12	63	20·5±6·0 (3-26)		
	18	46	20·2±7·0 (3-26)		
	24	26	21·5±3·9 (14-26)		
	>27	6	21·8±3·0 (19-26)		
**Pretreatment**					
no	-6	1	0·0	n=107	n=65
	0	131	4·9±7·5 (0-26)	6·0±4·9 (-7-17)	10·6±7·9 (-6-26)
	6	107	11·5±7·3 (0-26)		
	12	65	16·8±8·0 (2-26)		
	18	39	16·7±7·8 (2-26)		
	24	16	20·8±6·6 (3-26)		
	>27	3	19·0±11·3 (6-26)		
nusinersen	-6	95	9·2±8·2 (0-26)	n=120	n=105
	0	134	11·4±7·8 (0-26)	3·7±4·3 (-7-17)	5·1±6·0 (-6-23)
	6	122	15·1±7·2 (0-26)		
	12	107	16·8±7·1 (1-26)		
	18	86	18·3±6·5 (3-26)		
	24	53	18·6±6·2 (0-26)		
	>27	19	17·0±6·9 (5-26)		
risdiplam	-6	6	5·0±4·2 (1-13)	n=6	n=2
	0	10	7·9±5·2 (0-17)	6·7±4·3 (1-14)	12·5±0·7 (12-13)
	6	6	16·8±5·8 (11-26)		
	12	2	19·5±3·5 (17-22)		
	18	1	16·0		
	24	-	-		
	>27	-	-		
nusinersen+risdiplam	-6	1	1·0	n=1	-
	0	2	2·5±0·7 (2-3)	0·0	
	6	1	2·0		
	12	-	-		
	18	-	-		
	24	-	-		
	>27	-	-		

Supplementary Table 5: Influencing factors sex, presymptomatic diagnosis at treatment and *SMN2* copy number on HINE score. Data are scores depicted in mean±SD (range) unless otherwise specified. SMN=survival motor neuron gene. HINE= Hammersmith Infant Neurological Examination.

	Sum Sq	Mean Sq	NumDF	DenDF	F value	Pr(>F)
**ANOVA table saturated LME model**						
follow-up	1282·01548	427·338495	3	353·676897	12·5881958	7·7421E-08***
age at treatment	136·627074	34·1567686	4	246·915722	1·00616278	0·40488146
pretreatment	424·063068	424·063068	1	253·064639	12·4917109	0·00048585**
SMN2 copy number	595·836222	595·836222	1	293·118187	17·5516671	3·704E-05***
presymptomatic diagnosis	942·08741	942·08741	1	330·564871	27·7512578	2·4924E-07***
follow-up: age at treatment	1536·70837	128·059031	12	349·265562	3·77226056	2·1164E-05***
follow-up: pretreatment	138·681189	46·2270631	3	351·160719	1·36171987	0·25428268
follow-up: *SMN2* copy number	74·2270054	24·7423351	3	355·176807	0·72883993	0·53532825
follow-up: presymptomatic diagnosis	219·661192	73·2203974	3	353·074819	2·15686793	0·09282731
**ANOVA table backwards reduced LME model**						
follow-up	1215·6115	405·203833	3	360·196461	11·9998377	1·6637E-07***
age at treatment	167·100963	41·7752409	4	242·761145	1·23714552	0·29571801
pretreatment	594·902132	594·902132	1	220·709161	17·6176246	3·916E-05***
SMN2 copy number	826·389993	826·389993	1	229·077765	24·4729811	1·4587E-06***
presymptomatic diagnosis	904·4882	904·4882	1	326·800369	26·7858067	3·9769E-07***
follow-up: pretreatment	3414·77241	284·564367	12	354·461195	8·42718141	4·5356E-14***
follow-up: presymptomatic diagnosis	361·3701	120·4567	3	359·906424	3·56724376	0·01435733*

Supplemental Table 6: Linear mixed model of CHOP scores using Type III analysis of variance table with Satterthwaite's method.

	Sum Sq	Mean Sq	NumDF	DenDF	F value	Pr(>F)
**ANOVA table saturated LME model**						
follow-up	290·664919	145·332459	2	159·879837	14·3788405	1·8095E-06***
age at treatment	24·4793575	6·11983938	4	128·177842	0·60548204	0·65938617
pretreatment	31·3357199	31·3357199	1	123·686246	3·10028002	0·08075035
SMN2 copy number	189·985889	189·985889	1	124·463231	18·7967424	2·9707E-05***
presymptomatic diagnosis	109·925471	109·925471	1	131·129528	10·8757591	0·00125421**
follow-up: age at treatment	353·432155	44·1790194	8	147·260633	4·37096488	9·3175E-05***
follow-up: pretreatment	30·6869764	15·3434882	2	145·505631	1·51804746	0·22258934
follow-up: *SMN2* copy number	10·6691328	5·33456641	2	145·940543	0·52778904	0·59102943

follow-up: presymptomatic diagnosis	47·9460321	23·9730161	2	156·327538	2·37183198	0·09665978
**ANOVA table backwards reduced LME model**						
follow-up	430·02619	215·013095	2	156·289517	20·9557278	8·6565E-09***
age at treatment	21·4472023	5·36180058	4	129·061732	0·52257484	0·7192951
pretreatment	304·986821	304·986821	1	125·661953	29·7247979	2·5328E-07***
SMN2 copy number	93·722649	93·722649	1	132·467398	9·13444978	0·003012**
presymptomatic diagnosis	387·778499	48·4723124	8	153·338075	4·7242359	3·3968E-05***
follow-up: age at treatment	290·664919	145·332459	2	159·879837	14·3788405	1·8095E-06***

Supplemental Table 7: Linear mixed model of HFSME scores using Type III analysis of variance table with Satterthwaite's method.

	**Sum Sq**	**Mean Sq**	**NumDF**	**DenDF**	**F value**	**Pr(>F)**
**ANOVA table saturated LME model**						
follow-up	3527·91238	1763·95619	2	414·48214	217·480583	2·6167E-65***
age at treatment	445·422897	111·355724	4	278·929078	13·7292003	3·1163E-10***
pretreatment	20·3464646	20·3464646	1	280·872958	2·50854359	0·11435581
SMN2 copy number	285·357944	285·357944	1	286·953502	35·1821732	8·6212E-09***
presymptomatic diagnosis	200·937091	200·937091	1	292·669598	24·7738102	1·1023E-06***
follow-up: age at treatment	1271·14557	158·893196	8	409·314095	19·5901606	4·9896E-25***
follow-up: pretreatment	40·0636104	20·0318052	2	411·305106	2·4697488	0·08586007
follow-up: *SMN2* copy number	137·134984	68·5674922	2	414·366584	8·45378036	0·00025206***
follow-up: presymptomatic diagnosis	283·04296	141·52148	2	413·685009	17·4483778	5·3085E-08***
**ANOVA table backwards reduced LME model**						
follow-up	3826·15207	1913·07604	2	416·759213	234·103195	7·1005E-69***
age at treatment	794·903055	198·725764	4	279·481692	24·3180801	2·7804E-17***
SMN2 copy number	262·095237	262·095237	1	284·090772	32·0726052	3·6353E-08***
presymptomatic diagnosis	233·233867	233·233867	1	293·881706	28·5408382	1·8416E-07***
follow-up: age at treatment	1623·19604	202·899505	8	410·810849	24·828821	3·4742E-31***
follow-up: *SMN2* copy number	104·574869	52·2874347	2	414·626291	6·39841558	0·00183325**
follow-up: presymptomatic diagnosis	333·750016	166·875008	2	415·765423	20·4205017	3·4713E-09***

Supplemental Table 8: Linear mixed model of HINE scores using Type III analysis of variance table with Satterthwaite's method.
